# Wide Temperature All‐Solid‐State Ti_3_C_2_T_x_ Quantum Dots/L‐Ti_3_C_2_T_x_ Fiber Supercapacitor with High Capacitance and Excellent Flexibility

**DOI:** 10.1002/advs.202305991

**Published:** 2023-12-13

**Authors:** Juan He, Fuquan Ma, Wenpu Xu, Xuexia He, Qi Li, Jie Sun, Ruibin Jiang, Zhibin Lei, Zong‐Huai Liu

**Affiliations:** ^1^ Key Laboratory of Applied Surface and Colloid Chemistry (Shaanxi Normal University) Ministry of Education Xi'an 710062 P. R. China; ^2^ Shaanxi Key Laboratory for Advanced Energy Devices Xi'an 710119 P. R. China; ^3^ School of Materials Science and Engineering Shaanxi Normal University Xi'an 710119 P. R. China

**Keywords:** fiber supercapacitor, F‐MMT/PVA‐DMSO flexible hydrogel, Ti_3_C_2_T_x_ fiber electrode, wet spinning, wide temperature all‐solid‐state

## Abstract

Ti_3_C_2_T_x_ Quantum dots (QDs)/L‐Ti_3_C_2_T_x_ fiber electrode (Q_3_M_7_) with high capacitance and excellent flexibility is prepared by a wet spinning method. The assembled units Ti_3_C_2_T_x_ nanosheets (NSs) with large size (denoted as L‐Ti_3_C_2_T_x_) is obtained by natural sedimentation screen raw Ti_3_AlC_2_, etching, and mechanical delamination. The pillar agent Ti_3_C_2_T_x_ QDs is fabricated by an ultrasound method. Q_3_M_7_ fiber electrode gave a specific capacitance of 1560 F cm^−3^, with a capacity retention rate of 79% at 20 A cm^−3^, and excellent mechanical strength of 130 Mpa. A wide temperature all‐solid‐state the delaminated montmorillonite (F‐MMT)/Polyvinyl alcohol (PVA) dimethyl sulfoxide (DMSO) flexible hydrogel (DHGE) (F‐MMT/PVA DHGE) Q_3_M_7_ fiber supercapacitor is assembled by using Q_3_M_7_ fiber as electrodes and F‐MMT/PVA DHGE as electrolyte and separator. It showed a volume specific capacitance of 413 F cm^−3^ at 0.5 A cm^−3^, a capacity retention of 97% after 10 000 cycles, an energy density of 36.7 mWh cm^−3^ at a power density of 311 mW cm^−3^, and impressive capacitance and flexibility over a wide temperature range of −40 to 60 °C. This work provides an effective strategy for designing and assembling wide temperature all‐solid‐state fiber supercapacitors with optimal balance of capacitive performance and flexibility.

## Graphical abstract

Ti_3_C_2_T_x_ Quantum dots (QDs)/L‐Ti_3_C_2_T_x_ fiber electrode (Q_3_M_7_) with high capacitance and excellent flexibility is prepared by a wet spinning method, and all‐solid‐state symmetric fiber supercapacitor (F‐MMT/PVA DHGE Q_3_M_7_) with excellent energy storage in a wide temperature from ‐40 to 60 °C is assembled on the basis of the optimizing balance of capacitive performance and flexibility.

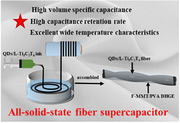

## 1 Introduction

With the rapid development of science and technology and the continuous improvement of people's living standards, the comfortable, lightweight, and multi‐functional wearable intelligent devices have been rapidly developed, and there is an urgent need to develop flexible energy storage system to match these devices.^[^ ^1^, ^2^, ^3^ ^]^ Fiber supercapacitors (FSCs) not only have large capacitance, high power density, long cycle life, but also have small size, light weight, good flexibility, and strong deformability.^[^ ^4^ ^]^ Therefore, FSCs can meet the high flexibility, weaving, and wearability requirements of the flexible devices such as wearable intelligent devices, making them ideal candidates for wearable intelligent device energy storage systems.^[^ ^5^ ^]^ Up to now, many functional fiber electrodes with excellent capacitance and good mechanical property have been prepared and the corresponding FSCs have been assembled.^[^ ^6^, ^7^, ^8^, ^9^, ^10^, ^11^ ^]^ Although FSCs have many advantages, their intrinsic low volume energy density and poor rate performance restrict their widespread commercial applications. Developing new functional fibers with impressive energy storage performance, high electrical conductivity, and strong mechanical property as well as assembling asymmetrical supercapacitors provides feasibility to resolve these questions.

2D layered materials have shown wide application promising in wearable and portable energy storage devices in recent years. They can be delaminated into nanosheets, and the delaminated nanosheets are ideal assembled units for fiber electrodes because of their large aspect ratio, high active specific surface area, excellent capacitance, liquid crystal phase characteristics, and so on.^[^ ^12^, ^13^, ^14^ ^]^ Ti_3_C_2_T_x_ is a typical 2D metal carbide, that can be delaminated to Ti_3_C_2_T_x_ nanosheets (NSs) in some suitable solvents. Some fiber electrode materials with relatively good storage energy performance and mechanical property have been prepared by using Ti_3_C_2_T_x_ NSs as the assembled units,^[^ ^15^, ^16^, ^17^, ^18^ ^]^ especially the Ti_3_C_2_T_x_ NSs nematic liquid crystal (LCs) at higher concentration shows obvious advantages in assembling functional fiber electrodes for wearable energy storage devices.^[^ ^19^ ^]^ Unfortunately, the prepared Ti_3_C_2_T_x_‐based hybrid fibers usually sacrifice capacitance to improve flexible property, and vice versa. How to achieve optimal the balance between capacitance and flexibility in preparing fiber electrodes is a problem that requires special attention during the preparation of Ti_3_C_2_T_x_ fiber electrodes.^[^ ^20^, ^21^, ^22^ ^]^ In general, some functional additives are introduced into the Ti_3_C_2_T_x_ NSs assembly system to improve the flexibility of the prepared fiber electrodes, and some Ti_3_C_2_T_x_‐based fiber electrodes with the optimal balance of the capacitive performance and flexibility have been prepared, such as TOCNFs/Ti_3_C_2_ fibers (2, 2, 6, 6‐tetramethylpi‐peridine‐1‐oxylradi‐cal (TEMPO)‐mediated oxidized cellulose nanofibril (TOCNFs)/Ti_3_C_2_ MXene),^[^ ^21^ ^]^ MXene/rGO fiber,^[^ ^22^ ^]^ MXene/PEDOS:PSS fibers (MXene/poly(3,4‐ethylene dioxythiophene):polystyrene sulfonate),^[^ ^23^ ^]^ and Ti_3_C_2_T_x_/ANF (Ti_3_C_2_T_x_/aramid nanofiber) hybrid fibers,^[^ ^24^ ^]^ etc. Although the flexibility of the prepared fiber electrodes has been obviously improved, their capacitance and rate performance degrade due to the absence or low capacitance of these added functional additives. Functional additives with good intrinsic capacitance may be a good choice for balancing the capacitance and flexibility property for fiber electrodes. In addition, FSCs devices assembled from the fiber electrodes give generally good device performance at room temperature, while they will severely degrade under severe cold or hot conditions.^[^ ^25^, ^26^, ^27^ ^]^ Therefore, it is an urgent need to develop wide‐temperature FSCs devices with an optimal balance between the capacitive performance and flexibility property and wide temperature usage range.

In the present work, two strategies were used to prepare Ti_3_C_2_T_x_‐based fiber electrode with excellent capacitive performance and good mechanical property. One is Ti_3_C_2_T_x_ NSs with large size (L‐Ti_3_C_2_T_x_ NSs) was obtained by natural sedimentation screen precursor Ti_3_AlC_2_,^[^ ^28^ ^]^ which were etched and followed by mechanically delaminated. The other was to use Ti_3_C_2_T_x_ quantum dots (QDs) with the same intrinsic property of Ti_3_C_2_T_x_ as pillar agent to reduce the easy recombination of L‐Ti_3_C_2_T_x_ NSs.^[^ ^29^ ^]^ The L‐Ti_3_C_2_T_x_ NSs were beneficial for improving the mechanical property of the prepared fiber electrode because the contact area and fiber forming force between the Ti_3_C_2_T_x_ NSs increase, while Ti_3_C_2_T_x_ QDs with the same intrinsic property of Ti_3_C_2_T_x_ NSs not only effectively prevent the assembling of L‐Ti_3_C_2_T_x_ NSs as the pillar agents, but also contribute effective capacitance. First, Ti_3_C_2_T_x_ QDs/L‐Ti_3_C_2_T_x_ NSs ink with a concentration of ≈15 mg mL^−1^ was obtained by mixing L‐Ti_3_C_2_T_x_ NSs dispersion and Ti_3_C_2_T_x_ QDs dispersion and followed by centrifuging. Second, Ti_3_C_2_T_x_ QDs/L‐Ti_3_C_2_T_x_ NSs ink was injected into a coagulated bath of 5 wt.% chitosan solution, pulled out from the coagulation bath and naturally dried to prepare Ti_3_C_2_T_x_ QDs/L‐Ti_3_C_2_T_x_ NSs fiber (Q_3_M_7_) with high capacitance and excellent flexibility. A series of Q_x_M_10‐x_ fibers were prepared by changing the amount of Ti_3_C_2_T_x_ QDs added while maintaining other preparation conditions. The prepared Q_3_M_7_ fiber electrode not only gave a specific capacitance of 1560 F cm^−3^ at 1 A cm^−3^ in 1 m H_2_SO_4_ electrolyte and a capacity retention rate of 79% at 20 A cm^−3^, but also showed good mechanical strength of 130 Mpa. By using Q_3_M_7_ fiber as electrodes and the delaminated montmorillonite (F‐MMT)/Polyvinyl alcohol (PVA) dimethyl sulfoxide (DMSO) flexible hydrogel (DHGE) (F‐MMT/PVA DHGE) as electrolyte and separator, a wide temperature all‐solid‐state F‐MMT/PVA DHGE Q_3_M_7_ fiber supercapacitor (F‐MMT/PVA DHGE Q_3_M_7_ FSC) was assembled. It showed a volume specific capacitance of 413 F cm^−3^ at 0.5 A cm^−3^ at room temperature, a capacity retention of 95% after 10 000 cycles, an energy density of 36.7 mWh cm^−3^ at a power density of 311 mW cm^−3^. In addition, the assembled device exhibited impressive capacitance and flexibility over a wide temperature range of −40 to 60 °C, showing that the assembled F‐MMT/PVA DHGE Q_3_M_7_ FSC displays broad application prospect in the field of portable/wearable electronic devices because of its high energy storage capacity, good flexibility and wide temperature characteristics.

## 2 Result and Discussion

**Figure 1:**
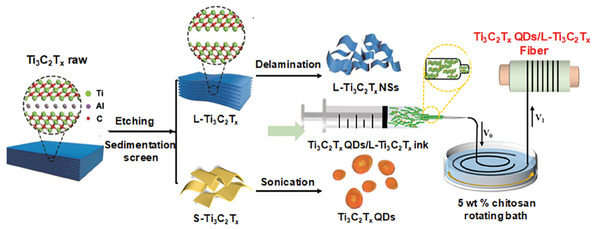
Preparation process schematic of Ti_3_C_2_T_x_ QDs/L‐Ti _3_C_2_T_x_ NSs fiber.

The preparation process of Ti_3_C_2_T_x_ QDs/L‐Ti_3_C_2_T_x_ NSs fiber electrode is shown in **Figure** **1**, and the detail information is described in the experimental section. The raw Ti_3_AlC_2_ shows irregular lamellar morphology with different sizes (Figure S1a, Supporting Information), while a relative uniform layered morphology L‐Ti_3_AlC_2_ with an average size of 10 µm is obtained by screening the raw Ti_3_AlC_2_ using natural sedimentation method (Figure S1b, Supporting Information).

**Figure 2:**
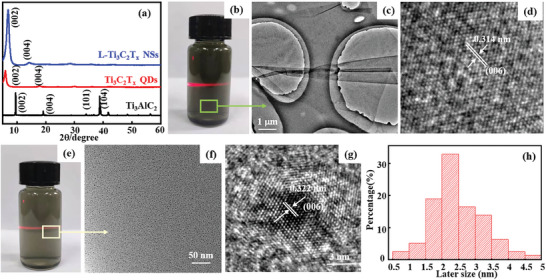
XRD patterns of raw L‐Ti_3_AlC_2_, the dried L‐Ti_3_C_2_T_x_ NSs, and Ti_3_C_2_T_x_ QDs (a). b) The suspension digital image, c) TEM image, and d) HR‐TEM image of L‐Ti_3_C_2_T_x_ NSs. e) The TEM image, f) suspension digital image, g) HR‐TEM image, and h) the size distribution plot of Ti_3_C_2_T_x_ QDs.

L‐Ti_3_AlC_2_ precursor shows a typical layered structure, after the Al layer is etched in a mixture solution of LiF and HCl. The Al diffraction peak at 39.9° in Ti_3_AlC_2_ disappears, indicating that the Al layer has been completely etched.^[^ ^30^ ^]^ Then the obtained multi‐layer L‐Ti_3_C_2_T_x_ is washed with deionized water by a suction filtration device until the pH of the suspension is ≈6, it was shaken by hand for 20 min, the multi‐layer L‐Ti_3_C_2_T_x_ is delaminated into L‐Ti_3_C_2_T_x_ NSs (**Figure** **2**a). The L‐Ti_3_C_2_T_x_ NSs suspension exhibits an obvious Tyndall effect due to its good hydrophilicity (Figure 2b), and the delaminated L‐Ti_3_C_2_T_x_ NSs shows a nanoplate morphology (Figure 2c). The size distribution statistics indicate that the average size of L‐Ti_3_C_2_T_x_ NSs is 7.6–9.5 µm according to the optical photo of Ti_3_C_2_T_x_ NSs (Figure S2a,b, Supporting Information), which is much larger than the average size of Ti_3_C_2_T_x_ NSs prepared by a conventional method.^[^ ^31^ ^]^ In addition, the thickness of L‐Ti_3_C_2_T_x_ NSs is ≈1.8 nm (Figure S2c,d, Supporting Information), which is slightly higher than that of the single‐layer Ti_3_C_2_T_x_ NSs due to the adsorption of other functional groups and H_2_O on the surface of L‐Ti_3_C_2_T_x_ NSs. Also, HR‐TEM image gives clear lattice fringes with a crystal plane spacing of 0.314 nm corresponding to (006) crystal plane of Ti_3_C_2_T_x_, indicating that the delaminated L‐Ti_3_C_2_T_x_ NSs still maintains a good crystal structure (Figure 2d). On the other hand, the delaminated L‐Ti_3_C_2_T_x_ NSs dispersion is sonicated for 8 h at a power of 480 W, Ti_3_C_2_T_x_ QDs with an average size of ≈2.5 nm and uniform distribution are fabricated (Figure 2e), and its suspension also shows obvious Tyndall effect (Figure 2f). Moreover, clear lattice fringes with a crystal plane spacing of 0.207 nm corresponding to the (106) crystal plane of Ti_3_C_2_T_x_ are observed, indicating the Ti_3_C_2_T_x_ QDs still have a good crystal structure and no oxidation behavior during the ultrasonic process of Ti_3_C_2_T_x_ NSs (Figure 2g). Moreover, the size distribution statistics indicate that the average size of Ti_3_C_2_T_x_ QDs is ≈2.3 nm (Figure 2h).

In order to assemble Ti_3_C_2_T_x_ fiber electrode with excellent capacitance and good mechanical property, L‐Ti_3_C_2_T_x_ NSs have been obtained by natural sedimentation screening, etching, and followed by mechanical delaminating. However, L‐Ti_3_C_2_T_x_ NSs are easily assembled, it will cause poor rate performance and mechanical property of Ti_3_C_2_T_x_ fiber electrode, thus Ti_3_C_2_T_x_ QDs with the same intrinsic property of Ti_3_C_2_T_x_ NSs are introduced into the interlayer of Ti_3_C_2_T_x_ NSs as pillar agent in the present work. The aspect ratio of L‐Ti_3_C_2_T_x_ NSs is ≈4000, a liquid crystal (LC) phase begins to appear at a 4.16 mg cm^−3^ of the L‐Ti_3_C_2_T_x_ dispersion concentration. According to a certain mass ratio, Ti_3_C_2_T_x_ QDs/L‐Ti_3_C_2_T_x_ NSs ink with a concentration of 15 mg mL^−1^ is obtained by mixing Ti_3_C_2_T_x_ NSs dispersion and Ti_3_C_2_T_x_ QDs suspension and followed by concentrating. Research results indicate that Ti_3_C_2_T_x_ NSs dispersion can form liquid crystalline (LCs) at higher concentration, and almost all Ti_3_C_2_T_x_ NSs in the ink are oriented to prepare highly oriented Ti_3_C_2_T_x_ fibers.^[^ ^32^ ^]^

**Figure 3:**
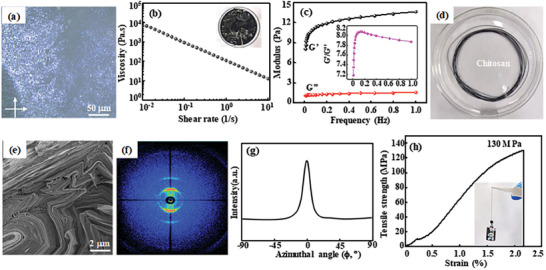
Rheological property of Ti_3_C_2_T_x_ QDs/L‐Ti_3_C_2_T_x_ (Q_3_M_7_) ink: a) Polarized optical microscopy (POM) image, b) the relationship between viscosity and shear rate (the optical image, inserted), c) the storage modulus (G’) and loss modulus (G’’) (the G’/G’’ ratio as a function of frequency in inset). The morphology and flexibility of Q_3_M_7_ fiber: d) the digital image prepared in 5 wt.% chitosan coagulation bath, e) the cross‐section FE‐SEM image, f) WAXS 2D scattering patterns, g) the azimuthal scattering plot at (002) along the ϕ direction in the region of ±90°, h) the tensile stress‐strain curve and the optical image holding a 20 g weight (inserted).

In general, a nematic phase is identified as threadlike topological defects called disclination, it is manifested by birefringent optical textures consisting of dark and bright brushes (known as schlieren textures) when it is observed under crossed polarizers.^[^ ^33^ ^]^ For Ti_3_C_2_T_x_ QDs/L‐Ti_3_C_2_T_x_ NSs ink with a concentration of 15 mg mL^−1^, an obvious schlieren texture is observed, indicating that a complete isotropic to anisotropic (I‐N) transition is occurred (**Figure** **3**a). Moreover, its viscosity gradually decreases with the increase of shear rate, and there is no agglomeration and phase separation for L‐Ti_3_C_2_T_x_ NSs in the dispersion medium (Figure 3b, inserted), suggesting a typical non‐Newtonian fluid behavior of shear thinning. This ink can be continuously extruded from the nozzle to form a stable and continuous composite fiber in a suitable coagulating bath. The storage modulus (G′) is greater than the loss modulus (G″) for the rheological property of Ti_3_C_2_T_x_ QDs/L‐Ti_3_C_2_T_x_ NSs ink, suggesting that this ink has gel‐like property (Figure 3c).^[^ ^34^ ^]^ In general, the ratio of G′ to G″ (G′/G″) can be used as an important parameter of LC Ti_3_C_2_T_x_ ink for spinnability,^[^ ^35^ ^]^ and G′/G″ values between 2.5 and 7.5 confirm that Ti_3_C_2_T_x_ ink can successfully form fibers through the wet spinning method.^[^ ^36^ ^]^ In the case of Ti_3_C_2_T_x_ QDs/L‐Ti_3_C_2_T_x_ NSs ink, a G′/G″ value of ≈7.5 is obtained, and it is relatively stable among 0.2–1.0 Hz, further indicating the typical viscoelastic gel‐like characteristics of Ti_3_C_2_T_x_ QDs/L‐Ti_3_C_2_T_x_ NSs ink. When Ti_3_C_2_T_x_ QDs/L‐Ti_3_C_2_T_x_ NSs ink with a concentration of 15 mg mL^−1^ is injected into a coagulated bath of 5 wt % chitosan solution at an injection rate of 30 mL h^−1^ using a syringe pump, L‐Ti_3_C_2_T_x_ NSs are agglomerated and Ti_3_C_2_T_x_ QDs are pillared into between L‐Ti_3_C_2_T_x_ NSs, and Ti_3_C_2_T_x_ QDs/L‐Ti_3_C_2_T_x_ NSs composite fiber with uniform and ultra‐long meter scale is prepared by the wet spinning method (Figure 3d). Ti_3_C_2_T_x_ QDs/L‐Ti_3_C_2_T_x_ NSs composite fiber shows a densely assemble morphology due to the slow coagulation rate in chitosan coagulated bath (Figure 3e) and is abbreviated as Q_3_M_7_ fiber, in which 3 is the added amount of Ti_3_C_2_T_x_ QDs and 7 is the amount of L‐Ti_3_C_2_T_x_ NSs added.

The 2D wide‐angle X‐ray scattering (WAXS) is used to characterize the structural feature of the prepared Q_3_M_7_ fiber. A representative scattering pattern including typical crescent ring can be observed, suggesting the presence of (002) peak of Ti_3_C_2_T_x_ in the obtained fiber (Figure 3f). After functionalization, Ti_3_C_2_T_x_ NSs and Ti_3_C_2_T_x_ QDs are respectively observed from TEM images (Figure S3, Supporting Information). In order to evaluate the orientation of Ti_3_C_2_T_x_ NSs relative to the fiber axis, the intensity along azimuth (ϕ) at the (002) peak is plotted to calculate the Herman's orientation factor (f) for assessing the orientation of polymer chains.^[^ ^37^ ^]^ The calculation result indicates that f value of the prepared Q_3_M_7_ fiber is ≈0.79, further suggesting the existence of ordered aligned Ti_3_C_2_T_x_ flakes inside the Q_3_M_7_ fiber (Figure 3g). In addition, a maximum tensile strength can reach up to 130 MPa for the prepared Q_3_M_7_ fiber (Figure 3h). Also it can independently pull up a 20 g weight (Figure 3h, inserted) and be bent at any angle without breaking and fracture after knotting (Figure S4, Supporting Information), suggesting that the obtained Q_3_M_7_ fiber possess good mechanical property due to its densely assemble of L‐Ti_3_C_2_T_x_ NSs. Moreover, EDS‐mapping images show that Ti, C and O elements are evenly distributed in Q_3_M_7_ fiber (Figure S5a, Supporting Information). XPS spectra indicates that Ti element in Q_3_M_7_ composite fiber mainly exists in Ti^3+^ and Ti^2+^ and no binding energy of TiO_2_ is observed, indicating L‐Ti_3_C_2_T_x_ NSs are not oxidized during the preparation process of fiber electrodes (Figure S5b, Supporting Information).^[^ ^38^ ^]^ At the same time, four peaks of O 1s are fitted at 530.0, 531.1, 532.2, and 533.0 eV, corresponding to Ti─O, C─Ti─O_x_, C─Ti─(OH)_x_, and H─O bonds, respectively (Figure S5c, Supporting Information). Furthermore, the N element does not appear in the spectrum and the binding energy of C─Ti─OH don't change, suggesting that on hydrogen bond is formed between ─OH and N^[^ ^39^ ^]^ and chitosan molecular is not residual in Q_3_M_7_ composite fiber. In addition, five de‐convoluted peaks centered at 281.6, 282.5, 284.6, 286.5, 288.4 eV reflect C─Ti, Ti─C─O, C─C, C─O, and O═C─O bonds, respectively (Figure S5d, Supporting Information).

Except for chitosan, different types of coagulation baths such as MgSO_4_/EtOH, NH_4_Cl/NH_4_OH, CaCl_2_/IPA are used to investigate their effects on the morphology and mechanical property of the obtained Ti_3_C_2_T_x_‐based fibers. The experimental results indicate that Ti_3_C_2_T_x_ QDs/L‐Ti_3_C_2_T_x_ NSs fiber with ultra‐long meter scale can also be prepared in these coagulation baths by a wet spinning method, suggesting Ti_3_C_2_T_x_ QDs/L‐Ti_3_C_2_T_x_ NSs ink has good spinnability (Figure S6, Supporting Information). Although Ti_3_C_2_T_x_‐based fibers with ultra‐long meter scale can be obtained, their morphology shows obvious difference. In compare with the dense assembly morphology of the prepared Q_3_M_7_ fiber in 5 wt.% chitosan coagulation bath, the loosely assembly morphology with different porous structure is obtained, suggesting a low relative mass density in the prepared fibers (Figure S7, Supporting Information). Because the hydrated ion radius such as Mg^2+^, NH_4_^+^, and Ca^2+^ ions is much smaller than that of the chitosan molecular chains, a rapid solvent exchange freezes L‐Ti_3_C_2_T_x_ NSs and thus generates a loose and open network structure in the obtained L‐Ti_3_C_2_T_x_‐based fiber. Soft solvent exchange causes a dense packing structure in the prepared Q_3_M_7_ fiber due to a large chitosan polymer chains.^[^ ^40^ ^]^

Moreover, the prepared Q_3_M_7_ fiber with dense packing structure in 5 wt.% chitosan coagulation bath entrusts better electrochemical property in compare with the obtained fibers with loose packing and open network structure in MgSO_4_/EtOH, NH_4_Cl/NH_4_OH, and CaCl_2_/IPA coagulation baths. Although the CV curve shape is similar to each other in the potential window of −0.4 to 0.4 V, the prepared Q_3_M_7_ fiber shows relatively larger CV integral area at 10 mV s^−1^ (Figure S8a, Supporting Information). The galvanostatic charge‐discharge curves (GCD) of the prepared fiber electrodes in four types of coagulation baths between −0.2 and 0.4 V at 1 A g^−1^ gives a triangular shape with a slight deformation, which reveals a good agreement with CV curves.^[^ ^41^ ^]^ According to the GCD curves, a specific capacitance of 1560 F cm^−3^ is obtained for Q_3_M_7_ fiber, which is larger than those of the other three fibers prepared in MgSO_4_/EtOH (805 F cm^−3^), NH_4_Cl/NH_4_OH (878 F cm^−3^), and CaCl_2_/IPA (912 F cm^−3^), respectively (Figure S8b, Supporting Information). In addition, Q_3_M_7_ fiber not only possesses good rate performance from 1 to 20 A g^−1^ (Figure S8c, Supporting Information), but also shows lower R_ct_ in the high frequency region, indicating that Q_3_M_7_ fiber electrode has good charge transfer reaction at the electrolyte‐electrode interface because its highly ordered structure could effectively shorten the diffusion distance of electrolyte (Figure S8d, Supporting Information, insert). Moreover, Q_3_M_7_ fiber electrode shows good mechanical properties in compare with those of the other three fibers (Figure S9, Supporting Information). The above results show that the Q_3_M_7_ fiber prepared by 5 wt.% chitosan coagulation bath possesses good electrochemical and mechanical properties due to the closer packing structure, thus 5 wt.% chitosan coagulation bath is suitable for preparing L‐Ti_3_C_2_T_x_‐based fiber.

In order to further investigate the effect of Ti_3_C_2_T_x_ QDs amounts on the electrochemical performance of L‐Ti_3_C_2_T_x_‐based fiber electrodes, a series of Q_x_M_10‐x_ fiber electrodes are prepared by keeping other preparation conditions as Q_3_M_7_ fiber, and only changing the added amount of Ti_3_C_2_T_x_ QDs in 5 wt.% chitosan coagulation bath, where x is the mass ratio of Ti_3_C_2_T_x_ QDs to L‐Ti_3_C_2_T_x_ NSs and they are 10%, 20%, 30%, and 40%, respectively. The XRD patterns show that the obtained fibers with different added amounts of Ti_3_C_2_T_x_ QDs have almost similar layered structures (Figure S10, Supporting Information), and they show similar dense assembly morphology (Figure S11, Supporting Information), suggesting that the addition of Ti_3_C_2_T_x_ QDs in the prepared fibers has almost no effects on the interlayer of the obtained fiber due to its small size.

**Figure 4:**
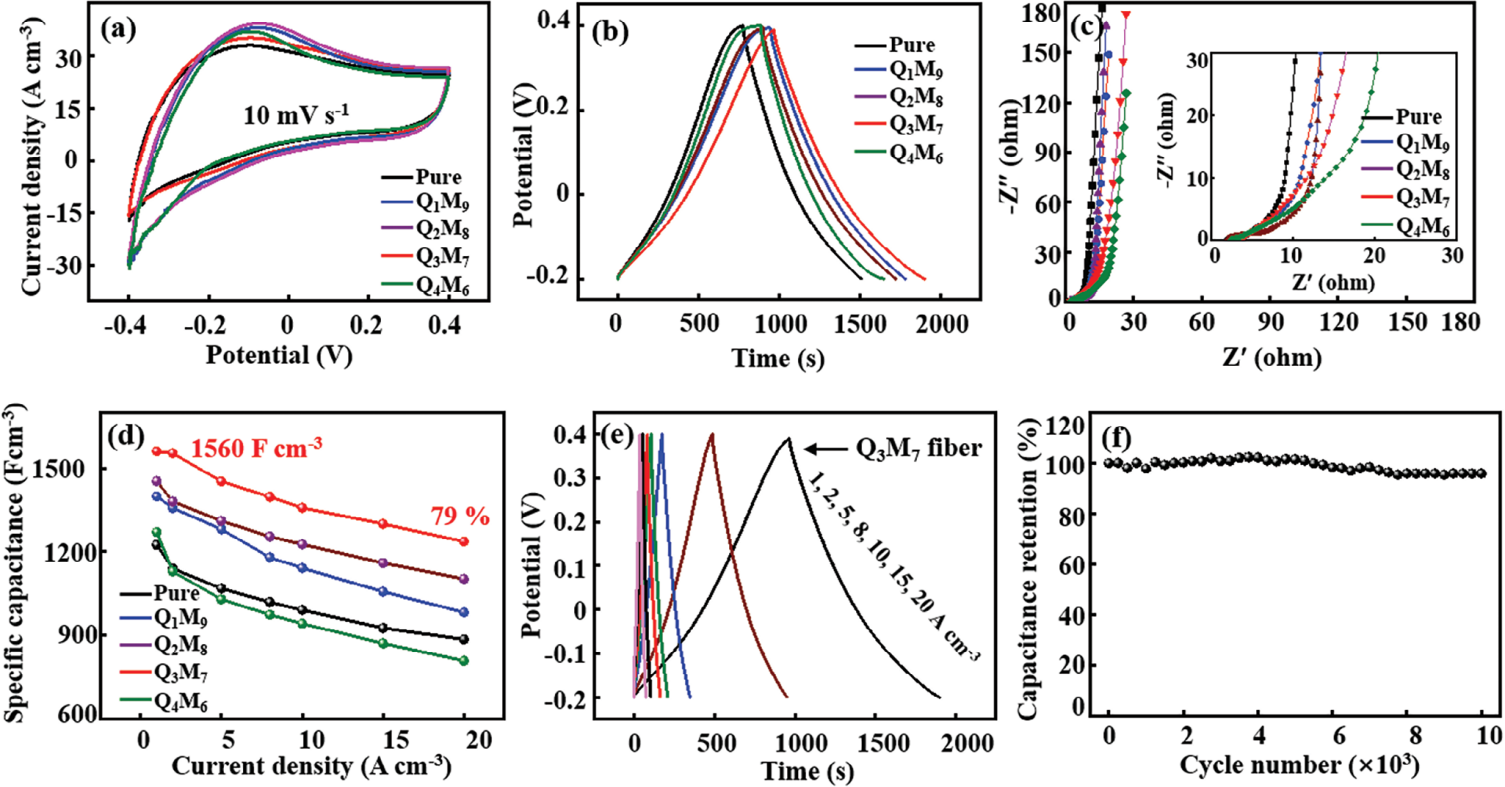
Electrochemical property and flexibility characterization of Ti_3_C_2_T_x_ QDs/L‐Ti_3_C_2_T_x_ fibers with different added amounts of Ti_3_C_2_T_x_ QDs: a) CV curves at 10 mV s^−1^, b) GCD curves at 1 A cm^−3^, c) Nyquist plots, d) capacitance as a function of the current density. GCD curves at 1–20 A cm^−3^ (e) and the capacity retention rate after 10 000 cycles at 50 mV s^−1^ (f) of Q_3_M_7_ fiber.

The electrochemical properties of the prepared Q_x_M_10‐x_ composite fiber electrodes are tested in 1 M H_2_SO_4_ electrolyte for investigating the effect of Ti_3_C_2_T_x_ QDs added amounts. In compare with CV curve of pure Ti_3_C_2_T_x_ fiber at 10 mV s^−1^, the integral area of CV curves from Q_1_M_9_ to Q_4_M_6_ composite fiber electrodes gradually increases, indicating that the added Ti_3_C_2_T_x_ QDs contribute to the specific capacitance for the prepared Q_x_M_10‐x_ composite fiber electrodes, which keeping a pair of redox peaks for the intercalation/deintercalation similar to H^+^ ions and surface redox reactions of Ti_3_C_2_T_x_ (**Figure** **4**a).^[^ ^42^ ^]^ According to the GCD curves, the pure L‐Ti_3_C_2_T_x_ fiber electrode shows a specific capacitance of 1158 F cm^−3^. In compare with the pure L‐Ti_3_C_2_T_x_ fiber electrode, a trend of capacitance first increasing and then decreasing is observed for Q_1_M_9_‐Q_4_M_7_ fiber electrodes, and Q_3_M_7_ fiber electrode displays a maximum specific capacitance of 1560 F cm^−3^, suggesting the optimal balance is needed for Ti_3_C_2_T_x_ QDs added amounts in prepared fiber electrodes (Figure 4b). The Nyquist plots indicate that all prepared fiber electrodes have small internal resistance (*R*_s_) and charge transfer resistance (*R*_ct_), suggesting that the introduction of Ti_3_C_2_T_x_ QDs do not significantly affect the ion dynamics for the assembled fibers (Figure 4c). Moreover, the prepared Q_1_M_9_‐Q_3_M_7_ fiber electrodes possess significant improvement rate performance form 1 A cm^−3^ to 20 A cm^−3^, the capacity retention is about for 70% of Q_1_M_9_, 75% of Q_2_M_8_, 79% of Q_3_M_7_, and 64% of Q_4_M_6_ in compare with 63% of the pure L‐Ti_3_C_2_T_x_ fiber electrode. Q_3_M_7_ fiber electrode shows the highest specific capacitance and optimal rate performance, suggesting that the addition of Ti_3_C_2_T_x_ QDs not only increase the specific capacitance of the prepared Q_x_M_10‐x_ fiber electrodes, but also improve their rate performance due to the pillar effect of Ti_3_C_2_T_x_ QDs in the interlayer of L‐Ti_3_C_2_T_x_ NSs (Figure 4d).

In compare with CV curve of Q_3_M_7_ fiber electrode with a pair of redox peaks corresponding to partial pseudocapacitance behavior at 10 mV s^−1^, the pair of redox peaks gradually disappear and the integral area of CV curves increases from 10 mV s^−1^ to 100 mV s^−1^, indicating that the pillar layered structure is more favorable to the electrolyte ion transport (Figure S12, Supporting Information). Corresponding to the CV curves of Q_3_M_7_ fiber electrode, the GCD curves keep high symmetric at all current densities with a negligible iR drop, suggesting that Q_3_M_7_ composite fiber electrode has good reversibility during the charging and discharging process (Figure 4e).^[^ ^43^ ^]^ After 10 000 cycles, Q_3_M_7_ composite fiber electrode gives a capacity retention of 96%, indicating that Q_3_M_7_ fiber electrode shows long cycle stability (Figure 4f). An efficient schematic with a cross‐sectional and lateral schematic diagrams of Q_3_M_7_ fiber is presented to support the relevance of QDs or associated functional composites in charge storage (Figure S13, Supporting Information), the quantum dots (QDs) distributed between Ti_3_C_2_T_x_ NSs not only effectively prevent the aggregation of NSs nanosheets, but also provide more active sites for the ion diffusion and improve the ion diffusion kinetics.

The charge storage kinetics of Q_3_M_7_ fiber electrode are further analyzed using CV curves, and the energy storage via capacitive behavior and diffusion‐controlled are investigated using equation *i* = a·v*^b^*, where *i*, *v*, a and b represent the peak current (mA cm^−3^), the scan rate (mV s^−1^), and the constant terms in the present work, respectively. The value of b can be determined from the slope of the straight‐line in the logarithm peaks current (log(i)) versus logarithm scan rate (log(v)) (Figure S14a, Supporting Information).^[^ ^38^ ^]^ In general, a diffusion‐controlled process is mainly for energy storage when *b* = 0.5, while a surface capacitive process is controlled for *b* = 1. For the anodic and cathodic peaks of Q_3_M_7_ fiber electrode, the b values are 0.67 and 0.58, respectively, suggesting that the pseudocapacitive behavior arises from protonation and changes in the oxidation states of Ti atoms, as well as the intercalation/de‐intercalation of electrolyte ions. Furthermore, the diffusive and capacitive component to the total capacitance can be further quantitatively based on equation *i*(V) = k_1_ *v*+k_2_ *v* ^0.5^, the capacitive contribution (green shaded area) to the total current reveals 38% basis on the CV curve of Q_3_M_7_ fiber at 10 mV s^−1^ (Figure S14b, Supporting Information).^[^ ^33^ ^]^ With increase the scan rates from 10 to 100 mV s^−1^, the capacitive contributions of Q_3_M_7_ fiber are increase, and the capacitive contribution of 78% is obtained at 100 mV s^−1^ due to H^+^ ion adsorption on Ti_3_C_2_T_x_ NSs and the weak Faraday reaction during the electrochemical process (Figure S14c, Supporting Information). In compare with other reported pure Ti_3_C_2_T_x_ fibers or Ti_3_C_2_T_x_‐based fiber electrodes, the prepared Q_3_M_7_ fiber electrode in the present work shows high volume specific capacitance, excellent rate performance, and good mechanical property (**Table** **1**),^[^ ^7^, ^20^, ^22^, ^24^, ^33^, ^36^, ^44^, ^45^, ^46^ ^]^ which means it is a superior Ti_3_C_2_T_x_‐based fiber electrode. This work provides a new reference for optimizing the balance between electrochemical property and mechanical property of fiber electrode.

**Table 1:** Performance comparison between Q_3_M_7_ fiber electrode and other reported Ti_3_C_2_T_x_‐based fiber electrodes.

i_3_C_2_T_x_ fibers	Conductivity [S cm^−1^]	Specific capacitance [F cm^−3^]	Rate capability	Tensile strength [MPa]	Electrolyte	References
Pure Ti_3_C_2_T_x_	≈7750	1265 (200 nm)	/	42 (3.1 µm)	1 m H_2_SO_4_	[7]
Ti_3_C_2_T_x_/PEDOT:PSS	1489	614	61% at 1 V s^−1^	58.1	1 m H_2_SO_4_	[20]
Ti_3_C_2_T_x_/RGO	72.3	341	37% at 10 A cm^−3^	132	1 m H_2_SO_4_	[22]
Ti_3_C_2_T_x_/ANF	1025	807	47% at 15 A cm^−3^	104	3 m H_2_SO_4_	[24]
O‐flat Ti_3_C_2_T_x_	7200	1360	49.6% at 1 V s^−1^	118	1 m MgSO_4_	[33]
Pure Ti_3_C_2_T_x_	7713	/	/	63.9	/	[36]
Pure Ti_3_C_2_T_x_	12504	/	/	344	/	[44]
Ti_3_C_2_T_x_/CNT	1142	295	19% at 1 V s^−1^	61	1 m H_2_SO_4_	[45]
Ti_3_C_2_T_x_/RGO	/	542	56% at 2 A cm^−3^	40	1 m H_2_SO_4_	[46]
Ti_3_C_2_T_x_/ L‐Ti_3_C_2_T_x_	2813	1560	79% at 20 A cm^−3^	123	1 m H_2_SO_4_	This work

In order to assemble all solid‐state flexible supercapacitors, flexible electrolyte has an important impact on the quality, volume, flexibility, and electrochemical window of the assembled devices.^[^ ^47^ ^]^ Although hydrogel electrolytes (HGE) have the advantages such as low cost, high deformability, high ionic conductivity similar to aqueous electrolytes, and flexibility of solid polymer electrolytes,^[^ ^48^ ^]^ the performance of the assembled HGE flexible supercapacitors (HGE FSC) is very vulnerable to changes in the surrounding environment due to the freezing and evaporating of water in the hydrogel electrolytes.^[^ ^49^ ^]^ Especially when the environment temperature is below 0 °C, the device performance for the assembled HGE FSC will significantly reduce due to the obvious reduction of the ionic conductivity, flexibility, and the close contact between electrolyte and electrode. Therefore, MMT flakes with thermodynamically stable are added to PVA gel electrolyte to improve the ionic conductivity by forming an ion channel. DMSO/H_2_O (*χ*_DMSO_ = 0.3) binary solvent system is used to reduce the antifreeze and long‐lasting moisture of PVA hydrogel in the present work.

**Figure 5:**
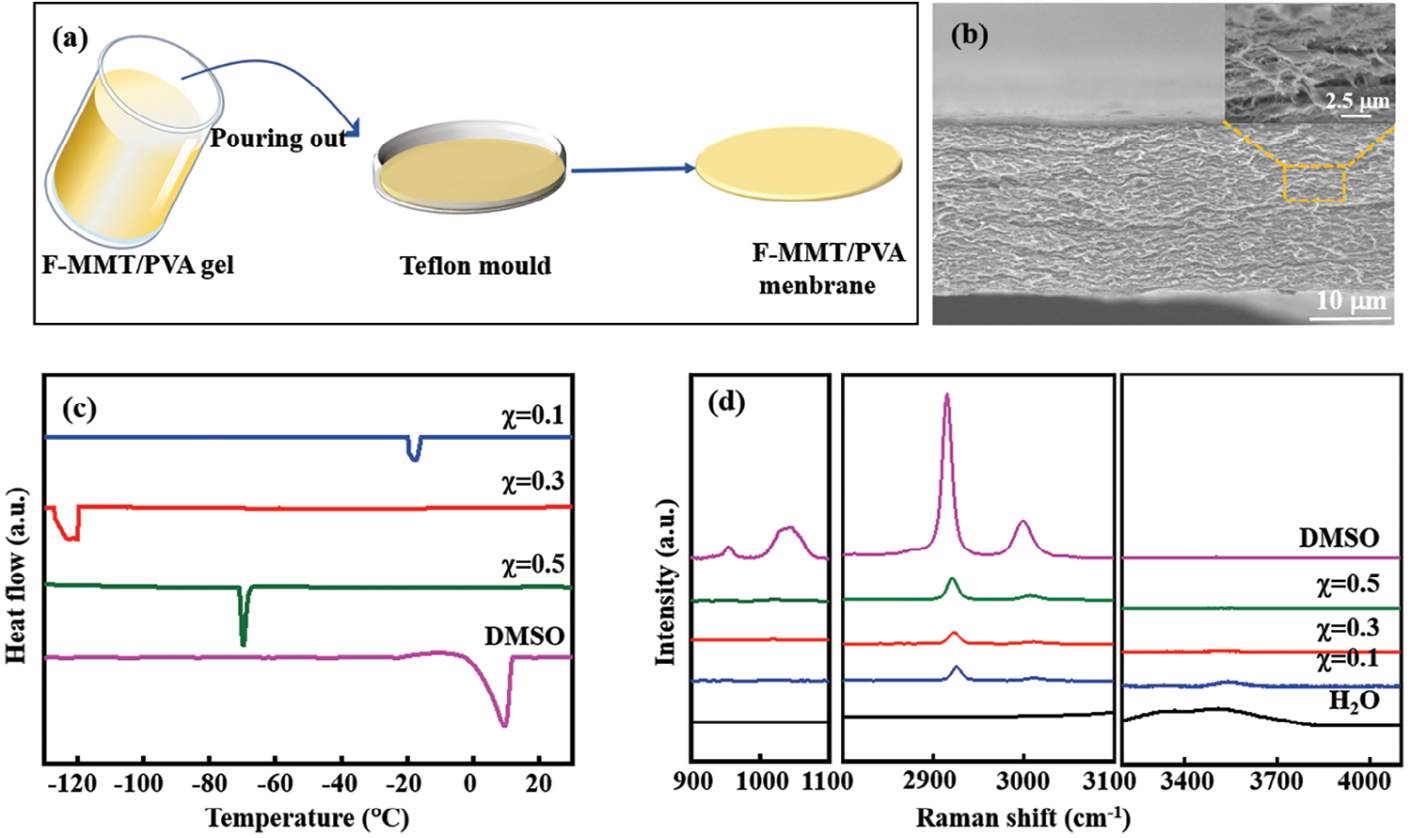
Fabrication process schematic a) and the cross‐section FE‐SEM image (insert, FE‐SEM image with high resolution) b) of F‐MMT/PVA hydrogel membrane. DSC curves c) and the local Raman spectra d) of DMSO/H_2_O binary solvent with different ratios of DMSO.

The preparation process of F‐MMT/PVA hydrogel membrane is shown in **Figure** **5**a with a 9:1 mass ratio of F‐MMT to PVA, and the detail information is described in the experimental part. A freeze‐thawing ultrasonic method is used to delaminate MMT bulk into few layer MMT NSs (F‐MMT NSs) with good hydrophilicity and large specific surface area and the delaminated F‐MMT NSs can be diffuse uniformly in PVA hydrogel.^[^ ^50^ ^]^ Raw MMT shows a blocky morphology (Figure S15a, Supporting Information), while F‐MMT NSs gives a layered thin morphology (Figure S15b, Supporting Information). Because F‐MMT NSs can be uniformly dispersed in PVA hydrogel without agglomeration or phase separation, F‐MMT/PVA hydrogel membrane with a 3D network structure can be observed from the cross‐sectional FE‐SEM image (Figure 5b, inserted), suggesting that the exfoliated F‐MMT is favorable to the formation of ion conductive pathway and promoting ionic dynamics in the electrochemical process. XRD spectra of F‐MMT NSs, PVA, and F‐MMT/PVA samples also support that the exfoliated F‐MMT NSs is successfully integrated into the PVA substrate. In compare with XRD patterns of F‐MMT NSs and PVA, F‐MMT/PVA hydrogel membrane mainly shows PVA diffraction peaks while F‐MMT NSs diffraction patterns are hardly observed, suggesting that the delaminated F‐MMT NSs are uniformly dispersed in PVA hydrogel and the reassembling of the delaminated F‐MMT NSs is not occurred (Figure S15c, Supporting Information). The elemental mappings show that Al, Si, O, and Mg elements are evenly distributed in the F‐MMT/PVA hydrogel membrane (Figure S16, Supporting Information). Moreover, the presence of inorganic cross‐linker agent F‐MMT NSs can effectively slow down the movement of PVA polymer chain segments at high temperature, which is beneficial to the increase of gel glass transition temperature and thus the obtained gel electrolyte has high temperature performance.

Moreover, DMSO with strong polarity and high boiling point can be miscible with water in any proportion, the hydrogen bonds between water molecules are weakened because it is a good hydrogen bonding receptor and thus the freezing point of the DMSO/H_2_O binary solvent system is significantly reduced.^[^ ^51^ ^]^ Differential scanning calorimetry (DSC) analysis indicates that the freezing point of DMSO/H_2_O binary solvent system can reach the lowest value of −120 °C when the molar ratio of DMSO is 0.3 (*χ*_DMSO_ = 0.3), which is significantly lower than other binary solvent systems with different DMSO ratios (DMSO = 0.1, 0.3, 0.5, 0.7) (Figure 5c), while both pure H_2_O and pure DMSO are frozen at −25 °C for 2 h (Figure S17a,b, Supporting Information). Raman spectroscopy shows that the O─H stretching mode (3300–3800 cm^−1^) has changed significantly with the increase of DMSO ratios, and the characteristic vibration band of sulfoxide (S═O) does not further change with DMSO ratios of 0.1 to 0.5 (Figure 5d),^[^ ^52^ ^]^ indicating that the hydrogen bonds between H_2_O molecules become weaker and a large amount of strong H‐bonds are formed between DMSO and water molecules with the addition of DMSO molecules. In addition, the F‐MMT/PVA hydrogel membrane is soaked in 1 m H_2_SO_4_ solution with *χ*_DMSO_ = 0.3 for 24 h to obtain F‐MMT/PVA‐DMSO hydrogel electrolyte, it is abbreviated as F‐MMT/PVA DHGE. It shows broader temperature adaptability from −40 to 60 °C, its ionic conductivity still maintains as high as 0.29 mS cm^−1^ even at an ultralow temperature of −40 °C, which is enough to meet the operational requirements of flexible FSCs. On the other hand, the ionic conductivity increases with the increases of the operating temperature, and it can reach 3.10 mS cm^−1^ when the operating temperature rises to 60°C without structure destruction (Figure S17c, Supporting Information).

**Figure 6:**
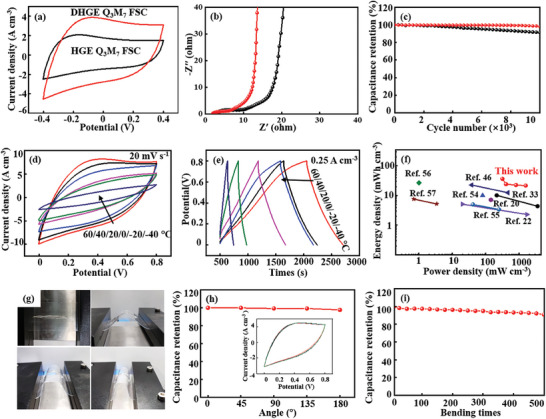
Comparison of the electrochemical property between F‐MMT/PVA DHGE Q_3_M_7_ FSC and PVA HGE Q_3_M_7_ FSC: a) CV curves at a scan rate of 20 mV s^−1^, b) Nyquist plots at 0.25 A cm^−3^, c) cycling stability after 10 000 cycles at 50 mV s^−1^. d) CV curves and e) GCD curves from −40 to 60 °C, ragone plot of F‐MMT/PVA DHGE Q_3_M_7_ FSC compared with other reported works (f), optical photographs at different bending angles (g), variation chart of capacity retention rate with bending angle (CV curves from 0° to 180°, inserted) (h), and capacity retention rate after 500 cyclic stretching i) of F‐MMT/PVA DHGE Q_3_M_7_ FSC.

A wide‐temperature all solid‐state F‐MMT/PVA DHGE Q_3_M_7_ fiber supercapacitor is assembled by using Q_3_M_7_ as electrodes and DMSO/H_2_O (*χ*_DMSO_ = 0.3) in 1 m H_2_SO_4_ as solvent, it is abbreviated as F‐MMT/PVA DHGE Q_3_M_7_ FSC. PVA HGE Q_3_M_7_ FSC is assembled with the same process without adding F‐MMT and DMSO in the electrolyte, and their electrochemical properties are measured by a two‐electrode system. The CV curves of the assembled two devices show quasi‐rectangular shape at 5 mV s^−1^, suggesting a typical double‐layer capacitance. Compared with the CV curve of PVA HGE Q_3_M_7_ FSC, the integrated area of F‐MMT/PVA DHGE Q_3_M_7_ FSC is significantly larger, indicating that F‐MMT/PVA DHGE Q_3_M_7_ FSC has a higher specific capacitance (**Figure** **6**a). GCD curves maintain good symmetry, indicating that the assembled two devices have fast reversible reaction characteristics. Meanwhile, a specific capacitance of 413 F cm^−3^ is obtained from its GCD curve for F‐MMT/PVA DHGE Q_3_M_7_ FSC, which is higher than that of 336 F cm^−3^ for PVA HGE Q_3_M_7_ FSC (Figure S18, Supporting Information). Moreover, F‐MMT/PVA DHGE Q_3_M_7_ FSC shows a smaller charge transfer resistance in the high frequency region in compare with PVA HGE Q_3_M_7_ FSC, suggesting it possesses lower diffusion resistance and higher charge transfer rate (Figure 6b). After 10 000 charge and discharge cycles at 50 mV s^−1^, the assembled F‐MMT/PVA DHGE Q_3_M_7_ FSC gives a capacity retention rate of 97%, it is significantly higher than that of PVA HGE Q_3_M_7_ FSC (87%) (Figure 6c).

For wide‐temperature all‐solid state flexible supercapacitor consisted of HGE, a wide temperature adaptability (from −40 to 60°C) and extraordinary resistance to mechanical damage (above 100 kg crushing) is very important.^[^ ^53^ ^]^ The assembled F‐MMT/PVA DHGE Q_3_M_7_ FSC shows a wide temperature adaptability, and can operate normally between −40 to 60 °C. At a scan rate of 20 mV s^−1^ in a wide temperature range of −40 to 60 °C, the CV curves of F‐MMT/PVA DHGE Q_3_M_7_ FSC maintains a nearly rectangular shape and the integral area enlarges with the increase of working temperature, suggesting that the assembled device can maintain continuous and stable working output in a wide temperature range of −40 to 60 °C (Figure 6d). This is mainly attributed to the increase of electrolyte ion mobility and migration rate with increase of the working temperature. The GCD curves still keep good symmetry at a current density of 0.25 A cm^−3^, indicating that the assembled F‐MMT/PVA DHGE Q_3_M_7_ FSC device has fast reversible reaction characteristics. Although the electrochemical performance reduces relatively with the increase of working temperature in the range of −40 to 60 °C, the assembled device still maintains high capacitance due to the increase of ion transfer resistance and the decrease of storage modulus (Figure 6e). These results show that the assembled F‐MMT/PVA DHGE Q_3_M_7_ FSC device has good energy storage performance in a wide temperature range of −40 to 60 °C. Moreover, the assembled F‐MMT/PVA DHGE Q_3_M_7_ FSC device shows good volumetric energy density. A maximum volumetric energy density of 36.7 mWh cm^−3^ is obtained when the power density is 311 mW cm^−3^. The comparison of Ragone plots between the F‐MMT/PVA DHGE Q_3_M_7_ FSC and the reported MXene‐based FSCs at room temperature indicates that F‐MMT/PVA DHGE Q_3_M_7_ FSC has a maximum energy density of 36.7 mWh cm^−3^, which is larger than commercial supercapacitors of 0.8 mWh cm^−3^ and also much higher than the thin‐film lithium battery of 0.3–10 mWh cm^−3^ (Figure 6f).^[^ ^54^, ^55^, ^56^, ^57^ ^]^ The four F‐MMT/PVA DHGE Q_3_M_7_ FSC in series can light up five red LED lights over a wide temperature range (Figure S19 and Video S1, Supporting Information), suggesting that the assembled flexible device shows some application scenarios.

In addition, F‐MMT/PVA DHGE Q_3_M_7_ FSC shows superior flexibility, which can be bent to different degrees (60°, 120°, and 180°) while the CV curves don't change significantly (Figure 6g,h). After 500 times of bending at 180°, the original capacitance retention is 95% (Figure 6i), suggesting that the electrochemical performance of the assembled device do not change significantly after bending and it has good mechanical property. Three factors contribute to the assembled F‐MMT/PVA DHGE Q_3_M_7_ FSC device show good electrochemical performance and flexibility. At first, F‐MMT/PVA/DMSO gel electrolyte provides ordered ion transport channels due to the special solvation of DMSO with water molecules, which reduces the solvation energy of electrolyte ions and speeds up the ion migration rate. Second, F‐MMT NSs promotes the gelation of the polymer and enhances the adhesion of the gel electrolyte, causing a close contact and reducing the interface resistance between gel electrolyte and electrode. Third, F‐MMT NSs increases the glass transition temperature of PVA gel, so that the gel electrolyte maintains good flow dynamics among wide temperature.

## 3 Conclusion 

L‐Ti_3_C_2_T_x_ NSs and Ti_3_C_2_T_x_ QDs are used to prepare Ti_3_C_2_T_x_ QDs/L‐Ti_3_C_2_T_x_ fiber electrode with high capacitance and excellent flexibility. L‐Ti_3_C_2_T_x_ NSs increase the contact area and fiber forming force between Ti_3_C_2_T_x_ NSs and improve the mechanical property of the prepared fiber electrode, while Ti_3_C_2_T_x_ QDs with the same intrinsic property effectively prevent the assembly of L‐Ti optimizing _3_C_2_T_x_ NSs and contribute effectively capacitance as pillar agents. Thus, the assembled Q_3_M_7_ fiber electrode shows a specific capacitance of 1560 F cm^−3^ at 1 A cm^−3^ in 1 m H_2_SO_4_ electrolyte, a capacity retention rate of 79% at 20 A cm^−3^, and an excellent mechanical strength of 130 Mpa. By providing ordered ion transport channels, speeding up the ion migration rate, maintaining good flow dynamics within the wide temperature from MMT/PVA/DMSO HGE, the optimizing balance of capacitive performance and flexibility, a wide temperature all‐solid‐state MMT/PVA DHGE Q_3_M_7_ FSC is assembled. The device displays a volume specific capacitance of 413 F cm^−3^ at 0.5 A cm^−3^ at room temperature, a capacity retention of 97% after 10 000 charging/discharging cycles, an energy density of 36.7 mWh cm^−3^ at a power density of 311 mW cm^−3^, and impressive capacitance and flexibility over a wide temperature range of −40 to 60 °C. This work provides an effective strategy for designing and assembling wide temperature all‐solid‐state FSSCs with optimal balance of capacitive performance and flexibility.

## 4 Experiment Section

### Materials

Ti_3_AlC_2_ raw was purchased from Beijing Forsman Technology Company. LiF (99.9%) was purchased from Aladdin. Polyvinyl alcohol (PVA, Mw: 89000‐98000) was purchased from Sigma–Aldrich Chemical Company. Dimethyl sulfoxide (DMSO, 99.7%), sulfuric acid (H_2_SO_4_, AR), and hydrochloric acid (HCl, AR) were purchased from Sinopharm Chemical Reagent Co., Ltd. Chitosan was purchased from Guoyao Company. Anhydrous magnesium sulfate (MgSO_4_, 99.99%) was purchased from Aladdin.

### Preparation of Q_3_M_7_ Fiber by Wet Spinning Technology

*Size Selection of L‐Ti_3_AlC_2_*: Ti_3_AlC_2_ particles with large size (L‐Ti_3_AlC_2_) was screened by natural settlement method. Ti_3_AlC_2_ (8 g, 200 mesh) was dispersed in 200 mL of deionized water (DI) by magnetic stirring for ≈10 min and stood 15 min. Then, the top dispersion containing suspended smaller Ti_3_AlC_2_ particles was decanted from the sediment and concentrated by centrifugation at 1500 rpm for 20 min, while the Ti_3_AlC_2_ particles with larger size were deposited in the bottom. The sedimentation process was repeated three times with the same conditions to fully select different size Ti_3_AlC_2_. The collected sediment was dried under vacuum at 50 °C for 6 h, Ti_3_AlC_2_ particles with large size was obtained and abbreviated as L‐Ti_3_AlC_2_ and for etching for Ti_3_C_2_T_x_ nanosheets with large size.

*Preparation of L‐Ti_3_C_2_T_x_ NSs and Ti_3_C_2_T_x_ QDs*: L‐Ti_3_AlC_2_ powder (4 g) was slowly added to an etching solution composed of 6.4 g LiF in HCl solution (9 m, 80 mL). The obtained suspension was reacted at 50 °C for 48 h to selectively etch the Al layer in Ti_3_AlC_2_ phase to form multi‐layer L‐Ti_3_C_2_T_x_ dispersion. The multi‐layer L‐Ti_3_C_2_T_x_ dispersion was washed with deionized water by the suction filtration device until the pH of the suspension was about neutral. Then the multi‐layer L‐Ti_3_C_2_T_x_ was again suspended in deionized water and the dispersion was shaken by hand for 20 min to delaminate multi‐layer L‐Ti_3_C_2_T_x_ into L‐Ti_3_C_2_T_x_ nanosheets. The L‐Ti_3_C_2_T_x_ nanosheets suspension was centrifuged at 2500 rpm for 30 min, the delaminated L‐Ti_3_C_2_T_x_ nanosheets were obtained and abbreviated as L‐Ti_3_C_2_T_x_ NSs.

Ti_3_C_2_T_x_ quantum dots were fabricated by an ultrasound method. In brief, the obtained Ti_3_C_2_T_x_ nanosheets dispersion (200 mL, 1 mg mL^−1^) was sonicated for 8 h at a power of 480 W, the resulting suspension was centrifuged at 11 000 rpm for 20 min, Ti_3_C_2_T_x_ quantum dots were suspended in upper dispersion while Ti_3_C_2_T_x_ nanosheets sediment were removed, the obtained Ti_3_C_2_T_x_ quantum dots were abbreviated as Ti_3_C_2_T_x_ QDs.

*Wet‐Spinning Preparation of Ti_3_C_2_T_x_ QDs/L‐Ti_3_C_2_T_x_ NSs (Q_3_M_7_) Fiber*: L‐Ti_3_C_2_T_x_ NSs (3 mg mL^−1^, 455 mL) and Ti_3_C_2_T_x_ QDs dispersion (1.3 mg mL^−1^, 150 mL) were first mixed, then it was concentrated by centrifugation at 16 000 rpm for 30 min, Ti_3_C_2_T_x_ QDs/L‐Ti_3_C_2_T_x_ NSs ink with a concentration of 15 mg mL^−1^ was obtained. Then Ti_3_C_2_T_x_ QDs/L‐Ti_3_C_2_T_x_ NSs ink was injected into a coagulated bath of 5 wt.% chitosan solution at an injection rate of 30 mL h^−1^ using a syringe pump, and the coagulation bath rotating speed was 40 mm s^−1^. The obtained fibers were pulled out from the coagulation bath and naturally dried, Ti_3_C_2_T_x_ QDs/L‐Ti_3_C_2_T_x_ NSs fiber was finally prepared, it was abbreviated as Q_3_M_7_ fiber. In addition, a series of Q_x_M_10‐x_ fibers were also prepared by keeping other preparation conditions with the same as above, and only by changing the added amount of Ti_3_C_2_T_x_ QDs, in which x was the added amounts of Ti_3_C_2_T_x_ QDs. The shear rate (γ, s^−1^) during spinning was estimated using the following equation:

(1) $$\gamma \;=\frac{4Q}{{\pi R}^{3}}\;\;\;\;$$

where Q was the flow rate (m^3^ s^−1^) and R was the inner radius of the needle (mm). The extrusion rates can be adjusted to obtain a constant shear rate during spinning.

### Preparation of MMT/PVA DHGE and PVA HGE

*Preparation of Few Layer Montmorillonite (MMT) Nanosheets*: The few layers montmorillonite (MMT) nanosheets were prepared by freezing/swelling and ultrasonic treatment. The MMT powder (5.0 g) was suspended in DI water (1000 mL) and stirred for 12 h, MMT nanosheets suspension was obtained. The obtained MMT nanosheets suspension was first frozen at −20 °C for 12 h, and then thawed at room temperature. This freezing/thawing process was repeated for three times, then the obtained MMT suspension was sonicated with a sonic tip at a power of 480 W for 0.5 h and centrifuged at 6000 rpm for 10 min to remove the undelaminated MMT. Freeze drying the dispersion to obtain few layer MMT nanosheets and abbreviate them as F‐MMT. Subsequently, F‐MMT (0.1 g) and PVA powder (0.9 g) were added to 10 mL H_2_O and stirred at 85 °C for 2 h to prepare F‐MMT/PVA gel. The F‐MMT/PVA gel was poured into a Teflon mold and vacuum dried for 4 h to obtain F‐MMT/PVA membrane. The F‐MMT/PVA membrane was soaked in 1 m H_2_SO_4_ using DMSO/H_2_O as solvent (χ = 0.3) for 12 h to prepare F‐MMT/PVA DHGE. PVA HGE was also prepared by the same method except for not using F‐MMT.

### Assembling of Wide‐Temperature All‐Solid‐State F‐MMT/PVA DHGE Q_3_M_7_ FSC

The prepared Q_3_M_7_ fiber was cut into two segments with a length of 2 cm. They were immersed in F‐MMT/PVA DHGE for 30 min and then dried for 30 min at room temperature. In order to ensure the uniform coating of F‐MMT/PVA DHGE, this process was repeated for three times. Then these two segments of Q_3_M_7_ fiber with a length of 2 cm were twisted tightly together, and soaked in 1 m H_2_SO_4_ solution using DMSO/H_2_O as solvent (χ_DMSO_ = 0.3) for 24 h, a wide‐temperature all‐solid‐state F‐MMT/PVA DHGE Q_3_M_7_ fiber symmetrical supercapacitor was assembled, it was abbreviated as F‐MMT/PVA DHGE Q_3_M_7_ FSC. In order to prevent the volatilization of water in gel electrolyte, the assembled F‐MMT/PVA DHGE Q_3_M_7_ FSC was wrapped with a seal film.

### Characterization

A Rigaku D X‐ray diffractometer (XRD) was used to study the crystal structure of the obtained samples, and the operating condition was at 40 kV and 15 mA by using Cu Kα radiation (*λ* = 1.541 Å). Raman spectra were recorded by Renishaw Raman InVia Confocal Microscope with the laser excitation was 532 nm, 5% of laser power. The morphology of samples obtained at different stages was observed by using scanning electron microscopy (SEM) on a Hitachi SU‐8020 field‐emission SEM system, transmission electron microscopy (TEM, JEM‐2100) and field‐emission scanning electron microscopy (FE‐SEM, SU2080). The EDS mapping of the sample was completed on the EDAX Genesis system. The optical image of L‐Ti_3_C_2_T_x_ NSs was taken by an optical microscope (BX53MRF‐S) and used Nanocsope to make the particle size distribution statistics. The composition and chemical state were determined by XPS (PHI Quantera II) spectrum and the binding energy calibration was referenced to C 1s at 284.8 eV. The freezing points of the electrolyte were tested using Q1000DSC (America TA Instruments) from ‐150 to 30 °C with a heating rate at 10 °C min^−1^ under air atmosphere. The 2D WAXS patterns of the obtained fibers were recorded by a Bruker Nanostar SAXS/WAXS system with an X‐ray wavelength of 1.5418 Å, and Pilatus 300 K was used as a 2D detector. The Herman's factors (f) was calculated to evaluate the Ti_3_C_2_T_x_ orientation according to the following equation, in which *I* was the structural unit and *Ф* was angle in reference direction:

(2) $$(\mathrm{co}s^{2}\varphi \;=\frac{\int I(\emptyset \sin\emptyset \cos\emptyset^{2}d\emptyset )}{\int I(\emptyset )\sin\emptyset d\emptyset }\;\;\;$$

(3) $$f\;=\;1-3(\mathrm{co}s^{2}\emptyset )$$

The stress–strain curves were tested by LDW‐5 tester from Shanghai Songton Instrument Manufacturing Co., and stretching rate was 0.5 mm min^−1^. Fourier transform infrared spectra (FT‐IR) were tested by using a Spotlight 400, Spectrum 100 FT‐IR Spectrometer, Perkin Elmer. The rheological performance of Ti_3_C_2_T_x_ inks was investigated using a rheometer6/20 (America TA Instruments, AR‐G2) with a cone‐shaped geometry (angle, 2°; diameter, 40 mm), and viscosity change as a function of shear rate was measured at shear rates between 0.01 and 10 s^−1^ using logarithmic steps. The viscoelastic properties of Ti_3_C_2_T_x_ inks were studied by measuring the elastic modulus (G’) and viscous modulus (G’’) as a function of frequency at a constant strain amplitude of 0.1%. Flexibility tests of the assembled F‐MMT/PVA DHGE Q_3_M_7_ FSC were carried out by using autonomous linear‐cycle stepper motor (DS‐4M001).

### Electrochemical Test

The electrochemical performance of the prepared fiber electrodes was evaluated by CHI660E electrochemical workstation in a three electrode system. Cyclic voltammetry (CV), galvanostatic charging‐discharging (GCD), and electrochemical impedance spectroscopy (EIS, 0.01 Hz–100 KHz) were measured in 1 M H_2_SO_4_ electrolyte. The specific capacitance (C_v_) was calculated from GCD curves by the following equation: *C_v_* = *I⋅*△*t*/(*v⋅*△*V*), where *C_v_* (F cm^−3^) is the specific capacitance, *I* (A) is discharging current, △*t* (s) is the discharging time, △*V* (V) is the potential window during the discharge process, and *v* (cm^−3^) was the volume of electrode materials, respectively.

The ionic conductivity of gel electrolyte was calculated in different temperatures according to the following equation: σ = L/R_e_⋅S, where L is the thickness of the F‐MMT/PVA gel electrolyte, *S* is the area and *R*_e_ is the bulk resistance. The bulk resistance was obtained from the electrochemical impedance (Nyquist plots). Different temperature electrochemical test of the assembled device was conducted in low temperature circulation system (DHJF‐4005) and the working temperature range is −40 to 60 °C. The volumetric energy density (*E_v_*) and the volumetric power density (*P_v_*) of the assembled device were calculated from the discharging curves at different current densities according to followed equations: *E_v_ = C_v_⋅△V* ^2^/7200, *P_v_* = 3600×*E_v_/△t*.

## Conflict of Interest

The authors declare no conflict of interest.

## Supporting information

Supporting Information

Supplemental Video 1

## Data Availability

The data that support the findings of this study are available in the supplementary material of this article.

## References

[1] M. R. Islam , S. Afroj , K. S. Novoselov , N. Karim , Adv. Sci. 2022, 9, 2203856. 10.1002/advs.202203856 PMC9631069 36192164

[2] K. Keum , J. W. Kim , S. Y. Hong , J. G. Son , S. S. Lee , J. S. Ha , Adv. Mater. 2020, 32, 2002180. 10.1002/adma.202002180 32930437

[3] X. Zhou , Z. Wang , T. Xiong , B. He , Z. Wang , H. Zhang , D. Hu , Y. Liu , C. Yang , Q. Li , M. Chen , Q. Zhang , L. Wei , Adv. Mater. 2023, 35, 2300576. 10.1002/adma.202300576 37042804

[4] Y. Zhang , J. Ding , B. Qi , W. Tao , J. Wang , C. Zhao , H. Peng , J. Shi , Adv. Funct. Mater. 2019, 29, 1902834.

[5] Y. Zhang , H. X. Mei , Y. Cao , X. H. Yan , J. Yan , H. L. Gao , H. W. Luo , S. W. Wang , X. D. Jia , L. Kachalova , J. Yang , S. C. Xue , C. G. Zhou , L. X. Wang , Y. H. Gui , Coord. Chem. Rev. 2021, 438, 213910.

[6] S. Chen , L. Qiu , H. M. Cheng , Chem. Rev. 2020, 120, 2811. 32073258 10.1021/acs.chemrev.9b00466

[7] J. Zhang , S. Uzun , S. Seyedin , P. A. Lynch , B. Akuzum , Z. Wang , S. Qin , M. Alhabeb , C. E. Shuck , W. Lei , E. C. Kumbur , W. Yang , X. Wang , G. Dion , J. M. Razal , Y. Gogotsi , ACS Cent. Sci. 2020, 6, 254. 32123744 10.1021/acscentsci.9b01217 PMC7047439

[8] L. Sheng , T. Wei , Y. Liang , L. Jiang , L. Qu , Z. Fan , Small 2017, 13, 1700371. 10.1002/smll.201700371 28417542

[9] Z. Jian , N. Yang , M. Vogel , S. Leith , A. Schulte , H. Schönherr , T. Jiao , W. Zhang , J. Müller , B. Butz , X. Jiang , Adv. Energy Mater. 2020, 10, 2002202.

[10] Z. Pan , J. Yang , Q. Zhang , M. Liu , Y. Hu , Z. Kou , N. Liu , X. Yang , X. Ding , H. Chen , J. Li , K. Zhang , Y. Qiu , Q. Li , J. Wang , Y. Zhang , Adv. Energy Mater. 2019, 9, 1802753.

[11] D. Yu , K. Goh , Q. Zhang , L. Wei , H. Wang , W. Jiang , Y. Chen , Adv. Mater. 2014, 26, 6790. 25182340 10.1002/adma.201403061

[12] T. F. Schranghamer , M. Sharma , R. Singh , S. Das , Chem. Soc. Rev. 2021, 50, 11032. 34397050 10.1039/d1cs00706h

[13] X. Xiao , H. Wang , P. Urbankowski , Y. Gogotsi , Chem. Soc. Rev. 2018, 47, 8744. 30302443 10.1039/c8cs00649k

[14] N. R. Glavin , R. Rao , V. Varshney , E. Bianco , A. A. Roy , E. Ringe , P. M. Ajayan , Adv. Mater. 2020, 32, 1904302. 10.1002/adma.201904302 31667920

[15] M. Naguib , M. Kurtoglu , V. Presser , J. Lu , J. Niu , M. Heon , L. Hultman , Y. Gogotsi , M. W. Barsoum , Adv. Mater. 2011, 23, 4248. 21861270 10.1002/adma.201102306

[16] B. Anasori , M. R. Luhatskaya , Y. Gogotsi , Nat. Rev. Mater. 2017, 2, 16098.

[17] J. Orangi , M. Beidaghi , Adv. Funct. Mater. 2020, 30, 2005305.

[18] M. R. Lukatskaya , O. Mashtalir , C. E. Ren , Y. Dall'agnese , P. Rozier , P. L. Taberna , M. Naguib , P. Simon , M. W. Barsoum , Y. Gogotsi , Science 2013, 341, 1502. 24072919 10.1126/science.1241488

[19] B. Wang , J. Liu , Y. Zhao , Y. Li , W. Xian , M. Amjadipour , J. Macleod , N. Motta , ACS Appl. Mater. Interfaces 2016, 8, 22316. 27529434 10.1021/acsami.6b05779

[20] J. Zhang , S. Seyedin , S. Qin , Z. Wang , S. Moradi , F. Yang , P. A. Lynch , W. Yang , J. Liu , X. Wang , J. M. Razal , Small 2019, 15, 1804732. 10.1002/smll.201804732 30653274

[21] W. T. Cao , C. Ma , D. S. Mao , J. Zhang , M. G. Ma , F. Chen , Adv. Funct. Mater. 2019, 29, 1905898.

[22] S. Seyedin , E. R. S. Yanza , J. M. Razal , J. Mater. Chem. A 2017, 5, 24076.

[23] Z. Li , Y. T. Xu , J. Hu , T. Wang , F. Q. Liu , H. Zhou , G. X. Chen , P. Lin , W. W. Zhao , J. J. Xu , H. Y. Chen , Sci China Chem 2023, 66, 578.

[24] Q. Liu , A. Zhao , X. He , Q. Li , J. Sun , Z. Lei , Z. H. Liu , Adv. Funct. Mater. 2021, 31, 2010944.

[25] Q. Liu , J. Zhou , C. Song , X. Li , Z. Wang , J. Yang , J. Cheng , H. Li , B. Wang , Energy Storage Mater. 2020, 24, 495.

[26] F. Mo , G. Liang , Q. Meng , Z. Liu , H. Li , J. Fan , C. Zhi , Energy Environ. Sci. 2019, 12, 706.

[27] Y. L. Sun , H. Y. Ma , X. Q. Zhang , B. Liu , L. Y. Liu , X. Zhang , J. Z. Feng , Q. N. Zhang , Y. X. Ding , B. J. Yang , L. T. Qu , X. B. Yan , Adv. Funct. Mater. 2021, 31, 2101277.

[28] N. Sun , Z. Guan , Q. Zhu , B. Anasori , Y. Gogotsi , B. Xu , Nano‐Micro Lett. 2020, 12, 89. 10.1007/s40820-020-00426-0 PMC7770857 34138104

[29] Z. Jin , C. Liu , Z. Liu , J. Han , Y. Fang , Y. Han , Y. Niu , Y. Wu , C. Sun , Y. Xu , Adv. Energy Mater. 2020, 10, 2000797.

[30] H. C. Huang , H. Su , H. T. Zhang , L. D. Xu , X. Chu , C. F. Hu , H. Liu , N. J. Chen , F. Y. Liu , W. Deng , B. N. Gu , H. P. Zhang , W. Q. Yang , Adv. Electron. Mater. 2018, 4, 1800179.

[31] A. A. Shamsabadi , A. P. Isfahani , S. K. Salestan , A. Rahimpour , B. Ghalei , E. Sivaniah , M. Soroush , ACS Appl. Mater. Interfaces 2020, 12, 3984. 31874026 10.1021/acsami.9b19960

[32] J. Zhang , N. Kong , S. Uzun , A. Levitt , S. Seyedin , P. A. Lynch , S. Qin , M. Han , W. Yang , J. Liu , X. Wang , Y. Gogotsi , J. M. Razal , Adv. Mater. 2020, 32, 2070180. 10.1002/adma.202001093 32309891

[33] S. Li , Z. Fan , G. Wu , Y. Shao , Z. Xia , C. Wei , F. Shen , X. Tong , J. Yu , K. Chen , M. Wang , Y. Zhao , Z. Luo , M. Jian , J. Sun , R. B. Kaner , Y. Shao , ACS Nano 2021, 15, 7821. 33834770 10.1021/acsnano.1c02271

[34] Y. Guo , X. Zhou , D. Wang , X. Xu , Q. Xu , Langmuir 2019, 35, 14481. 31622108 10.1021/acs.langmuir.9b02619

[35] S. Naficy , R. Jalili , S. H. Aboutalebi , R. A. Gorkin Iii , K. Konstantinov , P. C. Innis , G. M. Spinks , P. Poulin , G. G. Wallace , Mater. Horiz. 2014, 1, 326.

[36] W. Eom , H. Shin , R. B. Ambade , S. H. Lee , K. H. Lee , D. J. Kang , T. H. Han , Nat. Commun. 2020, 11, 2825. 32499504 10.1038/s41467-020-16671-1 PMC7272396

[37] H. Park , H. Park , H. Ahn , W. Lee , J. Park , B. J. Kim , D. K. Yoon , Chem. Mater. 2023, 35, 1355.

[38] C. H. Yang , Y. Tang , Y. P. Tian , Y. Y. Luo , Y. C. He , X. T. Yin , W. X. Que , Adv. Funct. Mater. 2018, 28, 1705487.

[39] G. R. Néstor , K. Masoomeh , M. G. Ángel , I. Francesc , Nanoscale Adv 2021, 3, 2793.36134196

[40] Q. Zhang , H. Lai , R. Fan , P. Ji , X. Fu , H. Li , ACS Nano 2021, 15, 5249. 33617227 10.1021/acsnano.0c10671

[41] M. Zhou , A. Gallegos , K. Liu , S. Dai , J. Wu , Carbon 2020, 157, 147.

[42] X. P. Mu , D. H. Wang , F. Du , G. Chen , C. Z. Wang , Y. J. Wei , Y. Gogotsi , Y. Gao , Y. Dall'agnese , Adv. Funct. Mater. 2019, 29, 1902953.

[43] H. C. Huang , J. Q. He , Z. X. Wang , H. T. Zhang , L. Jin , N. J. Chen , Y. T. Xie , X. Chu , B. N. Gu , W. L. Deng , W. Q. Yang , Nano Energy 2020, 69, 104431.

[44] H. Shin , W. Eom , K. H. Lee , W. Jeong , D. J. Kang , T. H. Han , ACS Nano 2021, 15, 3320. 33497182 10.1021/acsnano.0c10255

[45] X. Zhao , J. Zhang , K. Lv , N. Kong , Y. Shao , J. Tao , Carbon 2022, 200, 38.

[46] X. Zhou , Y. Qin , X. He , Q. Li , J. Sun , Z. Lei , Z. H. Liu , ACS Appl. Mater. Interfaces 2020, 12, 11833. 32023025 10.1021/acsami.9b21874

[47] Z. Wang , L. Wang , W. Jiang , X. Jian , F. Hu , Sci. China Mater. 2023, 66, 3129.

[48] Y. Yan , S. Duan , B. Liu , S. Wu , Y. Alsaid , B. Yao , S. Nandi , Y. Du , T. W. Wang , Y. Li , X. He , Adv. Mater. 2023, 35, 2211673. 10.1002/adma.202211673 36932878

[49] F. Mo , G. Liang , Q. Meng , Z. Liu , H. Li , J. Fan , C. Zhi , Energy Environ. Sci. 2019, 12, 706.

[50] T. Chen , Y. Yuan , Y. Zhao , F. Rao , S. Song , Langmuir 2019, 35, 2368. 30645941 10.1021/acs.langmuir.8b04171

[51] Q. Nian , J. Wang , S. Liu , T. Sun , J. Chen , Angew. Chem., Int. Ed. 2019, 58, 16994. 10.1002/anie.201908913 31541502

[52] V. M. Wallace , N. R. Dhumal , F. M. Zehentbauer , H. J. Kim , J. Kiefer , J. Phys. Chem. B 2015, 119, 14780. 26509778 10.1021/acs.jpcb.5b09196

[53] A. Chaichi , G. Venugopalan , R. Devireddy , C. Arges , M. R. Gartia , ACS Appl. Energy Mater. 2020, 3, 5693.

[54] T. Zhao , D. Yang , S. M. Hao , T. Xu , M. Zhang , W. Zhou , Z. Z. Yu , J. Mater. Chem. A 2023, 11, 1742.

[55] F. Ma , L. Li , C. Jia , X. He , Q. Li , J. Sun , R. Jiang , Z. Lei , Z. H. Liu , J. Colloid Interf. Sci. 2023, 643, 92. 10.1016/j.jcis.2023.04.014 37054547

[56] K. A. S. Usman , J. Zhang , S. Qin , Y. Yao , P. A. Lynch , P. Mota‐Santiago , M. Naebe , L. C. Henderson , D. Hegh , J. M. Razal , J. Mater. Chem. A 2022, 10, 4770. 10.1002/marc.202200114 35344626

[57] C. Yu , Y. Gong , R. Chen , M. Zhang , J. Zhou , J. An , F. Lv , S. Guo , G. Sun , Small 2018, 14, 1801203. 10.1002/smll.201801203 29943392

