# Supplementary material for: Wide Temperature All‐Solid‐State Ti3C2Tx Quantum Dots/L‐Ti3C2Tx Fiber Supercapacitor with High Capacitance and Excellent Flexibility
Source: Adv Sci (Weinh). 2023 Dec 13;11(7):2305991. doi: 10.1002/advs.202305991 (PMC10870075; doi:10.1002/advs.202305991)
Supplement: Supplementary file 1 — Supporting Information [file ADVS-11-2305991-s001.pdf]

ADVANCED SCIENCE
Open Access

Supporting Information

for *Adv. Sci.*, DOI 10.1002/advs.202305991

Wide Temperature All-Solid-State $Ti_3C_2T_x$ Quantum Dots/L-$Ti_3C_2T_x$ Fiber Supercapacitor with High Capacitance and Excellent Flexibility

Juan He, Fuquan Ma, Wenpu Xu, Xuexia He, Qi Li, Jie Sun, Ruibin Jiang, Zhibin Lei and Zong-Huai Liu**

Supporting information

Wide temperature all solid state $Ti_3C_2T_x$ quantum dots/L-$Ti_3C_2T_x$ fiber supercapacitor with high capacitance and excellent flexibility

Juan He[§ abc], Fuquan Ma[§ abc], Wenpu Xu[abc], Xuexia He*[bc], Qi Li[bc], Jie Sun[bc], Ruibing Jiang[bc], Zhibin Lei[abc], Zong-Huai Liu*[abc]

[a]Key Laboratory of Applied Surface and Colloid Chemistry (Shaanxi Normal University), Ministry of Education, Xi'an, 710062, P. R. China

[b]Shaanxi Key Laboratory for Advanced Energy Devices, Xi'an, 710119, P. R. China

[c]School of Materials Science and Engineering, Shaanxi Normal University, Xi'an, 710119, P. R. China

*Correspondence should be addressed to:

Zong-Huai Liu

School of Materials Science and Engineering, Shaanxi Normal University, Xi'an, Shaanxi,710062, P. R. China

Tel: 86-29-81530706

Fax: 86-29-81530702

E-mail: zhliu@snnu.edu.cn

Author Contributions

§ These authors contributed equally

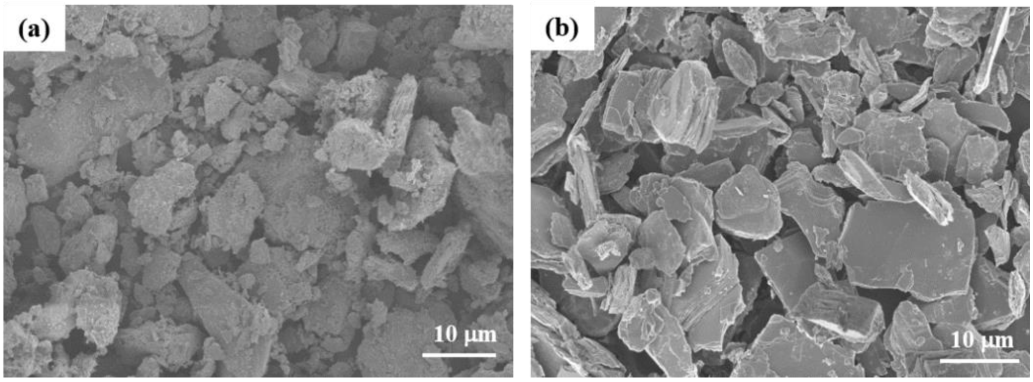

Figure S1. SEM images of raw Ti_3AlC_2 (a) and L-Ti_3AlC_2 after size selection (b).

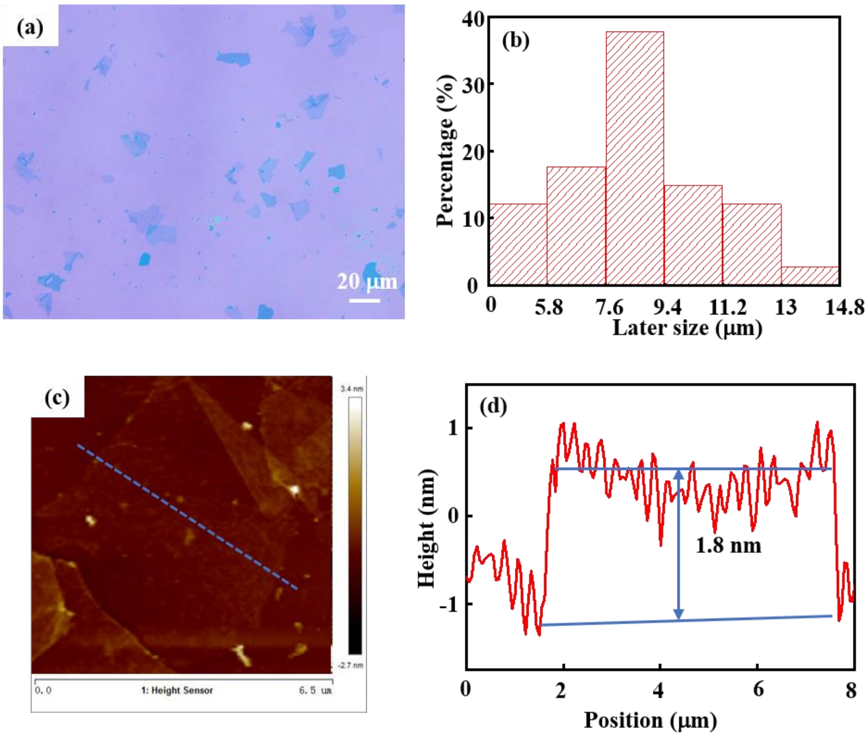

Figure S2. Optical image (a), the size distribution statistics (b), AFM image (c), and the thickness distribution (d) of L-$Ti_3C_2T_x$ NSs.

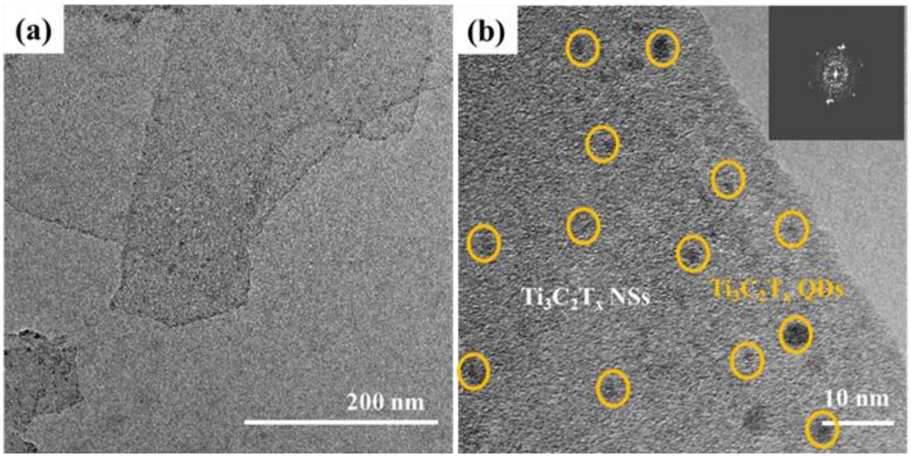

Figure S3. TEM image of Q_3M_7 fiber: (a) low magnification and (b) high magnification.

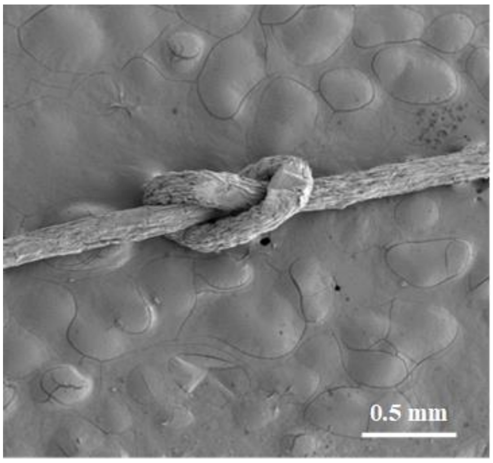

Figure S4. The weavability and FE-SEM image of the knotted Q_3M_7 fiber.

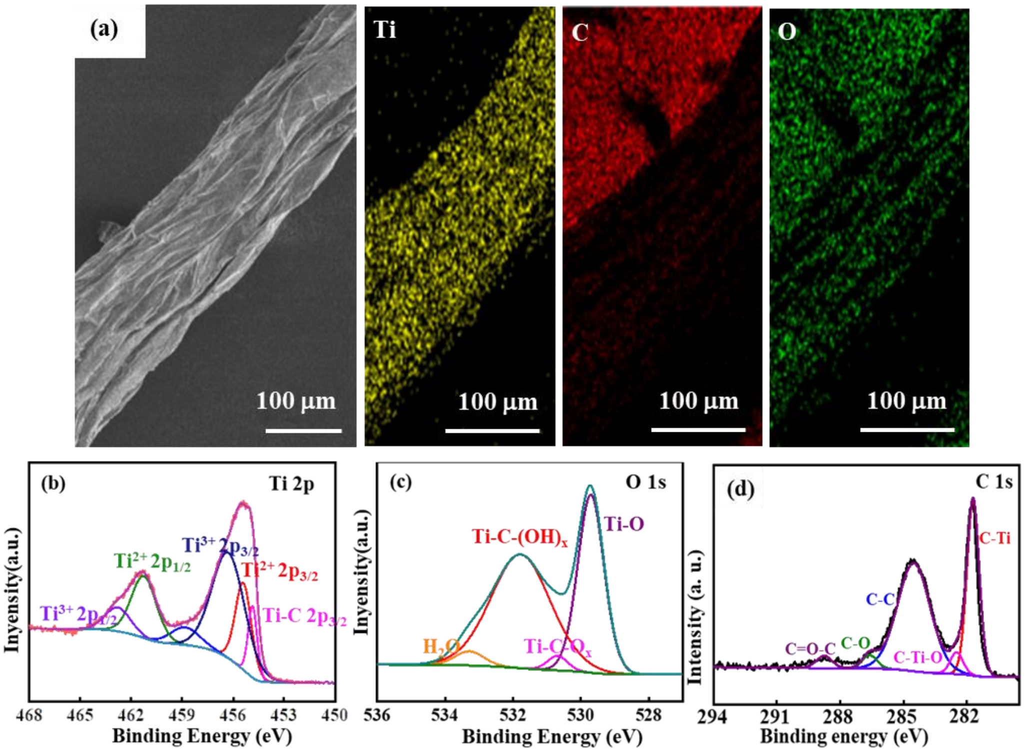

Figure S5. EDS mapping images (a) and XPS spectra of Q_3M_7 fiber: Ti 2p (b), O 1S (c), and C 1s (d).

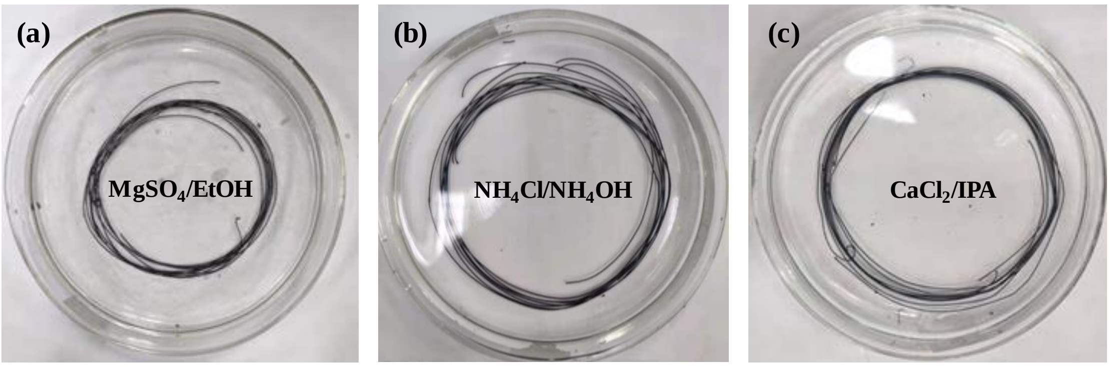

Figure S6. Optical photographs of the $Ti_3C_2T_x$-based fibers prepared in other three coagulation baths.

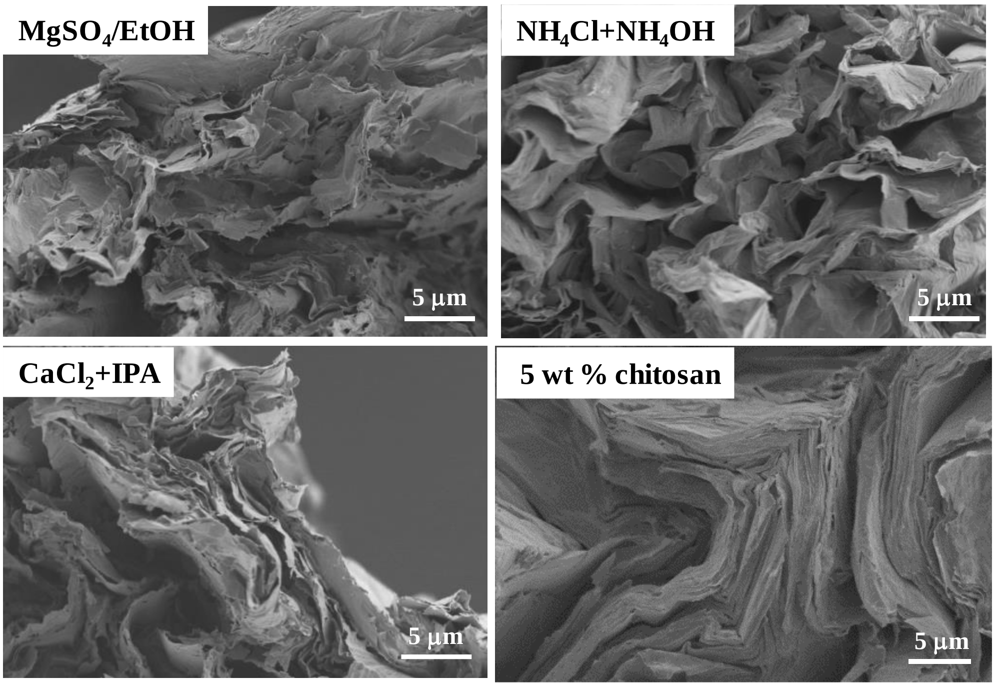

Figure S7. SEM images of L-$Ti_3C_2T_x$ based fibers prepared in different coagulation baths.

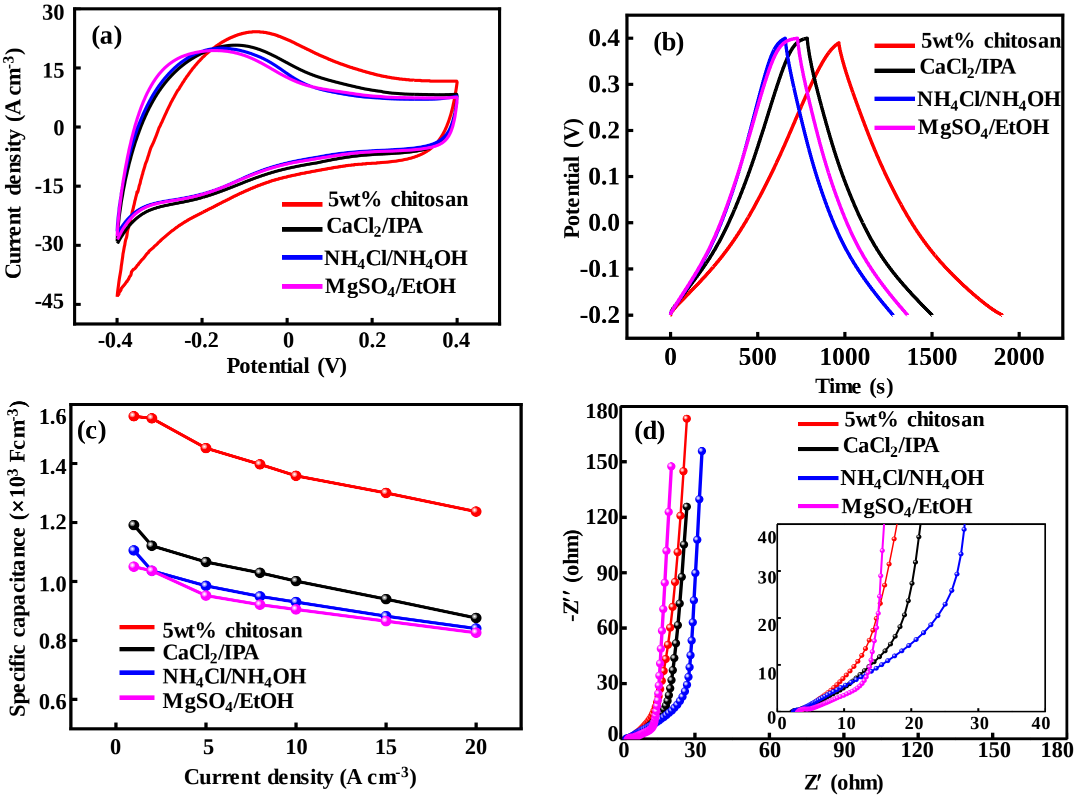

Figure S8. Electrochemical properties of L-$Ti_3C_2T_x$-based fibers prepared in different coagulation baths: CV curves at 10 mV s^{-1} (a), GCD curves at 1A cm^{-3} (b), capacitance as a function of the current density (c), and Nyquist plots (d).

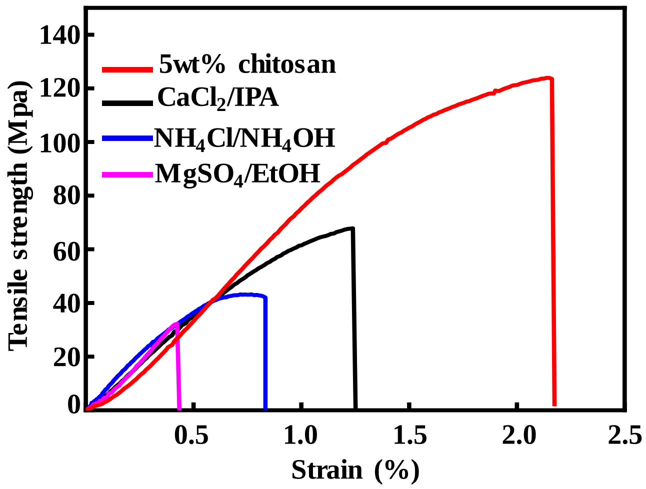

Figure S9. Stress-strain curves of L-$Ti_3C_2T_x$-based fibers prepared in different coagulation baths.

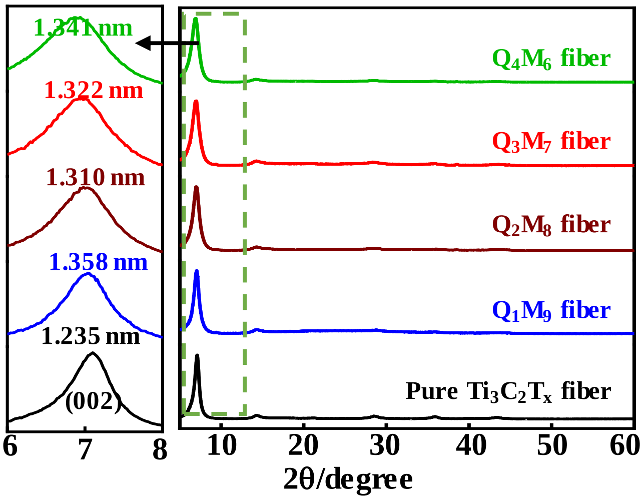

Figure S10. XRD patterns of the pure $Ti_3C_2T_x$ fiber and L-$Ti_3C_2T_X$-based fibers obtained with different added amounts of $Ti_3C_2T_x$ QDs.

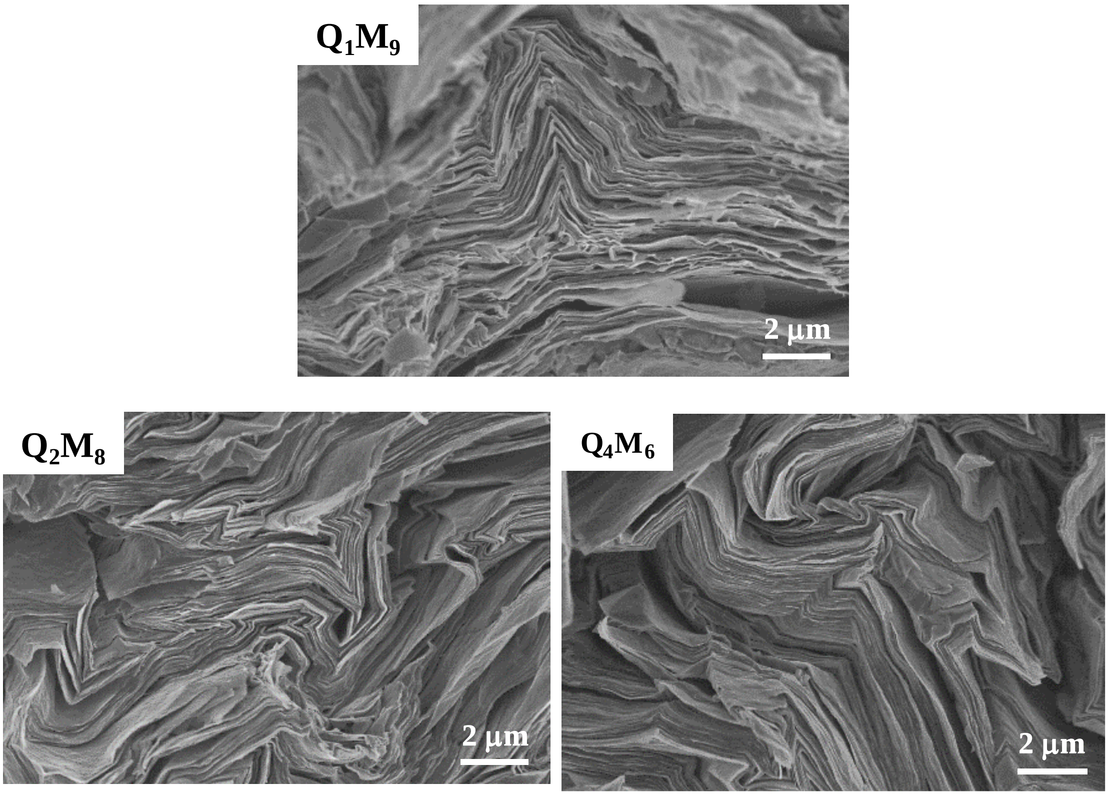

Figure S11. SEM images of L-$Ti_3C_2T_X$-based fibers obtained with different added amounts of $Ti_3C_2T_x$ QDs.

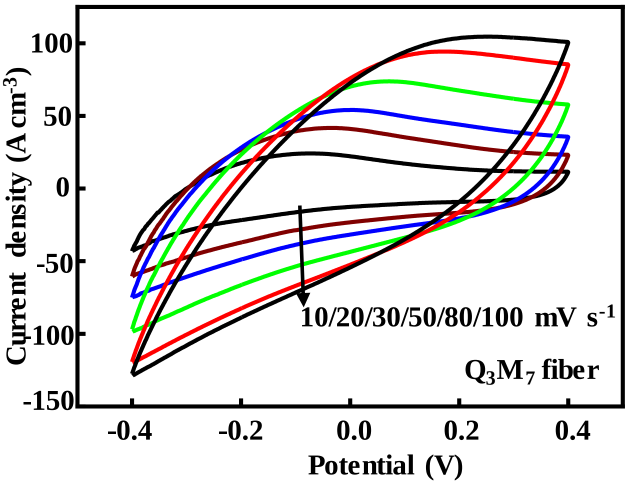

Figure S12. CV curves of Q_3M_7 fiber at different scan rates.

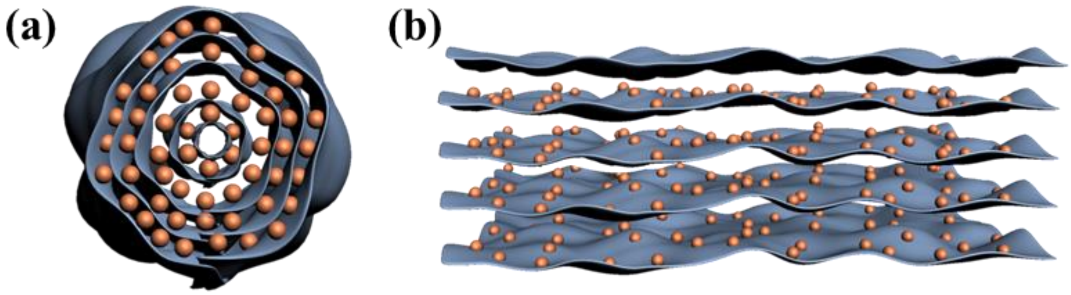

Figure S13. An efficient schematic with a cross-sectional and lateral schematic diagrams of Q_3M_7 fiber

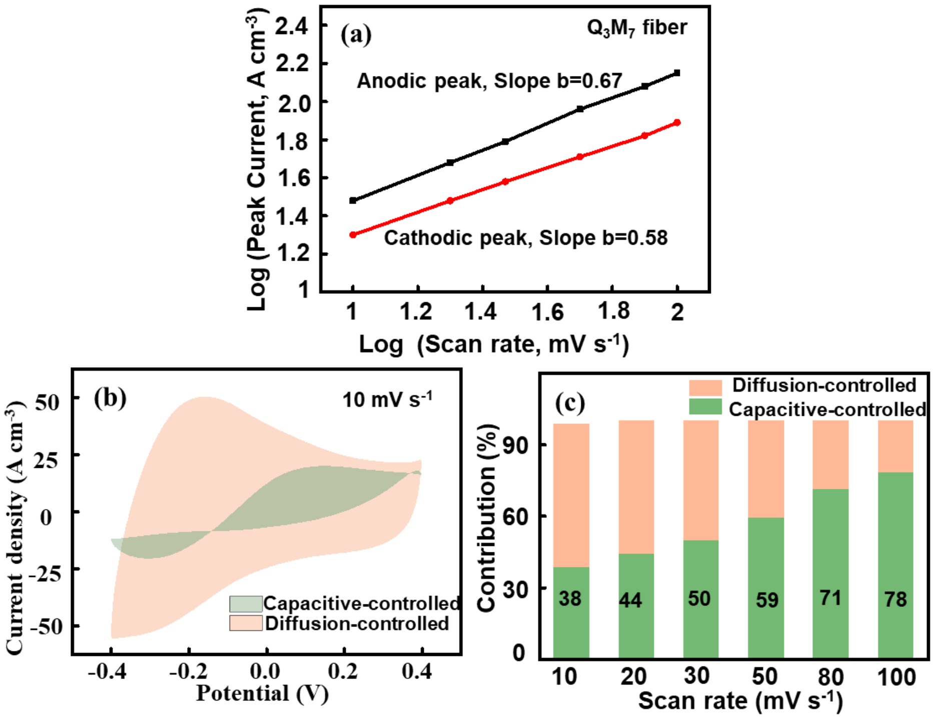

Figure S14. Log (scan rate) versus log (peak current) curves for the anodic and cathodic peaks (a) of Q_3M_7. Capacitive and diffusion limited contributions to total charge storage at 10 mV s^{-1} and the capacitive contribution to the total current marked with green shaded area (b) and the contribution ratio of capacitive (green) and diffusion limited contribution (light pink) of Q_3M_7 at various scan rates (c).

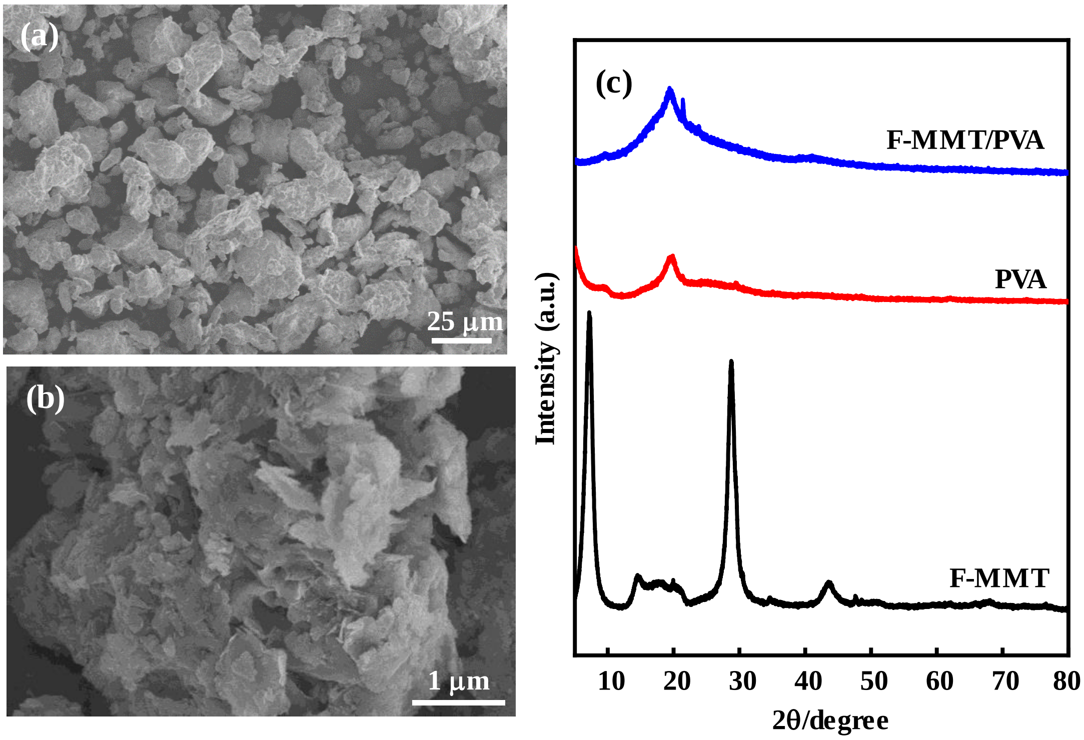

Figure S15. SEM images of raw MMT (a) and the delaminated F-MMT NSs (b). XRD patterns (c) of F-MMT NSs, PVA, and F-MMT/PVA.

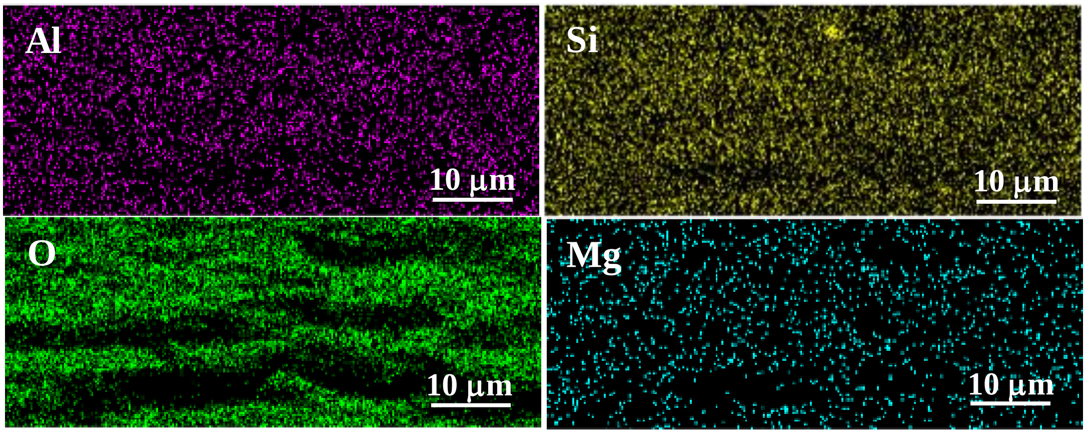

Figure S16. EDS-mapping images of F-MMT/PVA gel membrane.

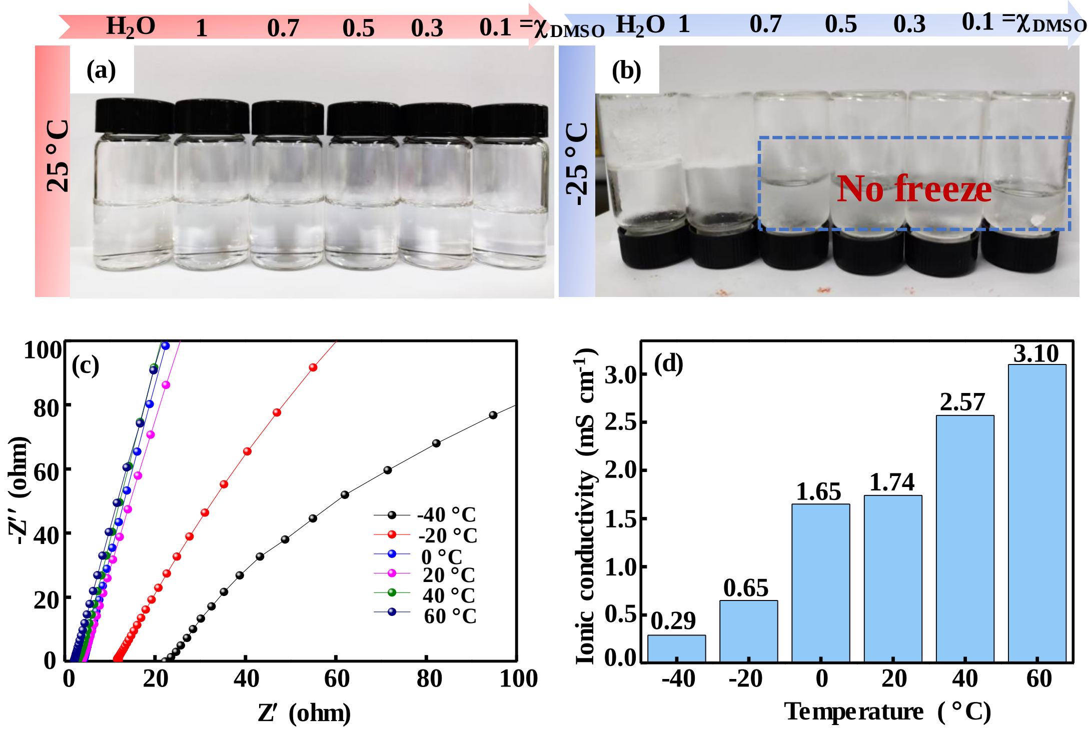

Figure S17. Optical images of DMSO/H_2O binary solvent systems composed with different DMSO amounts (χ_{DMSO}=0.1, 0.3, 0.5, 0.7 and 1) placed at 25 ºC (a) and -25 ºC (b) for 2 hours, the ionic conductivity (c, d) of MMT/PVA/DMSO (χ_{DMSO}=0.3) gel electrolyte between -40 ºC to 60 ºC.

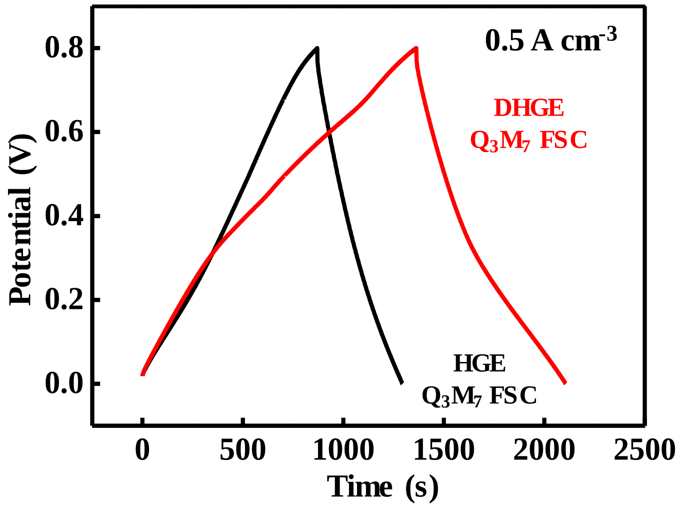

Figure S18. GCD curves of PVA HGE Q_3M_7 FSCs and F-MMT/PVA DHGE Q_3M_7 FSCs at 0.5 A cm^{-3}.

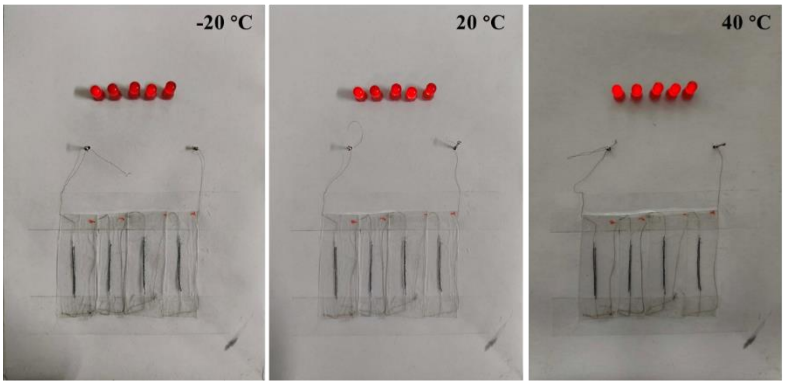

Figure S19. Light up a LED lamp at different temperatures for F-MMT/PVA DHGE Q_3M_7 FSC.
